# The moa footprints from the Pliocene – early Pleistocene of Kyeburn, Otago, New Zealand

**DOI:** 10.1080/03036758.2023.2264789

**Published:** 2023-11-14

**Authors:** Kane Fleury, Emma Burns, Marcus D. Richards, Kevin Norton, Stephen Read, Rachel Wesley, R. Ewan Fordyce, Klaus Wilcken

**Affiliations:** a Tūhura Otago Museum, Dunedin, New Zealand; b Department of Geology, University of Otago, Dunedin, New Zealand; c School of Geography, Environment and Earth Science, Victoria University of Wellington, Wellington, New Zealand; d Aukaha, Dunedin, New Zealand; e Australian Nuclear Science and Technology Organisation, Sydney, New Zealand

**Keywords:** Trackway, moa footprint‌, Dinornithiformes, cosmogenic nuclide dating, Pliocene‌, Maniototo Conglomerate

## Abstract

In March, 2019, a trackway of seven footprints was found at a riverbank outcrop of Maniototo Conglomerate Formation in the Kyeburn River, Central Otago, South Island, New Zealand. In this study, we describe this first known occurrence of moa (Dinornithiformes) footprints to be found and recovered in Te Waipounamu/South Island. Footprints of the trackway were ∼46 mm deep, 272–300 mm wide and 260–294 mm in length. An associated separate footprint was 448 mm wide and 285 mm long. Cosmogenic nuclide dating of adjacent overlying beds from the same formation establishes a mean minimum age of burial age for the tracks of 3.57 Ma (+1.62/−1.18 Ma) with a mode of 2.9 Ma, which we interpret to be Late Pliocene, with a conservative age range of Pliocene to Early Pleistocene. The trackway maker is identified as a moa from the Emeidae family, probably from the genus *Pachyornis*, with a mean mass of 84.61 kg that was travelling at a speed of 2.61 kmh^−1^. The single adjacent footprint was made by an individual from the family Dinornithidae, most likely from the genus *Dinornis* with an estimated mass of 158 kg. These moa footprints represent the second earliest fossil record of moa.

## Introduction

There were nine species of moa (Dinornithiformes) in New Zealand (Bunce et al. 2009; Checklist Committee 2022). Seven of these species of moa were found in South Island and four in Te Ika a Māui/North Island (Worthy and Scofield 2012; Checklist Committee 2022). The species within Dinornithiformes varied from the size of a large turkey (e.g. coastal moa, *Euryapteryx curtis curtis*) up to the 3 m tall female giant moa (*Dinornis* spp.)*.* Some species showed high amounts of sexual dimorphism with females being considerably larger than the males (Bunce et al. 2003; Hyunen et al. 2003; Bunce et al. 2009; Olson and Turvey 2013). Moa were important mahika kai (natural food source) species to Māori until their extinction approximately 600 years ago (Holdaway and Jacomb 2000; Rawlence and Cooper 2013; Perry et al. 2014). Despite their early demise, the importance of moa to Māori was well preserved in Te Reo and whakataukī (Wehi et al. 2018).

The first known moa footprints were found near the mouth of the Tūranganui River in Tairawhiti Gisborne in 1866 (Cockburn-Hood 1875; Gillies 1872; Williams 1872). Since then, several other sporadic finds have been made across the North Island (see review by Lockley et al. 2007) but moa footprints have only recently been found in the South Island. These include the Kyeburn footprints described here as well as those found in Paeroa, south of Timaru, South Canterbury in 2022 (Srinivasa 2022). Moa footprints have received considerable attention in the world of ichnology as the feet of the female giant moa have left the largest footprints of any bird that has ever been found (Lockley et al. 2007). The footprints of moa are easily compared to abundant skeletal material from moa as well as to extant species of Palaeognath such as emu, cassowary and ostrich. Information derived from biomechanical studies of modern ratites is often then applied to moa (Brassey et al. 2013; Farlow et al. 2013).

Footprints preserved in sediments are termed trace fossils or ichnofossils and are a specific kind of fossil. Ichnofossils are defined as the fossil record of an activity, or of a track maker that does not contain any remains of the animal itself. Vertebrate ichnofossils can include; tracks, trails, burrows, nests, feeding traces like bite marks, coprolites, gastroliths, and regurgitated pellets (Hasiotis et al. 2007). With moa it is possible to identify the probable track maker with a high level of confidence providing the geological age of the deposition of the sediments is well refined (Lockley and Harris 2010). Moa have an exquisite fossil record over the past 60,000 years (Worthy and Holdaway 2002; Rawlence et al. 2012), and a well resolved taxonomy (Checklist Committee 2022), allowing a high degree of comparison to known families, genera and species (Farlow et al. 2013).

Here, we present a description and specific dimensions of the footprints and trackway from Kyeburn as well as estimates of the size, weight and speed of travel of the moa across the trackway. We also hypothesise what kind of moa left these impressions. Additionally, we provide a minimum age of the footprints via cosmogenic nuclide dating.

### Discovery and excavation

**Figure 1.**
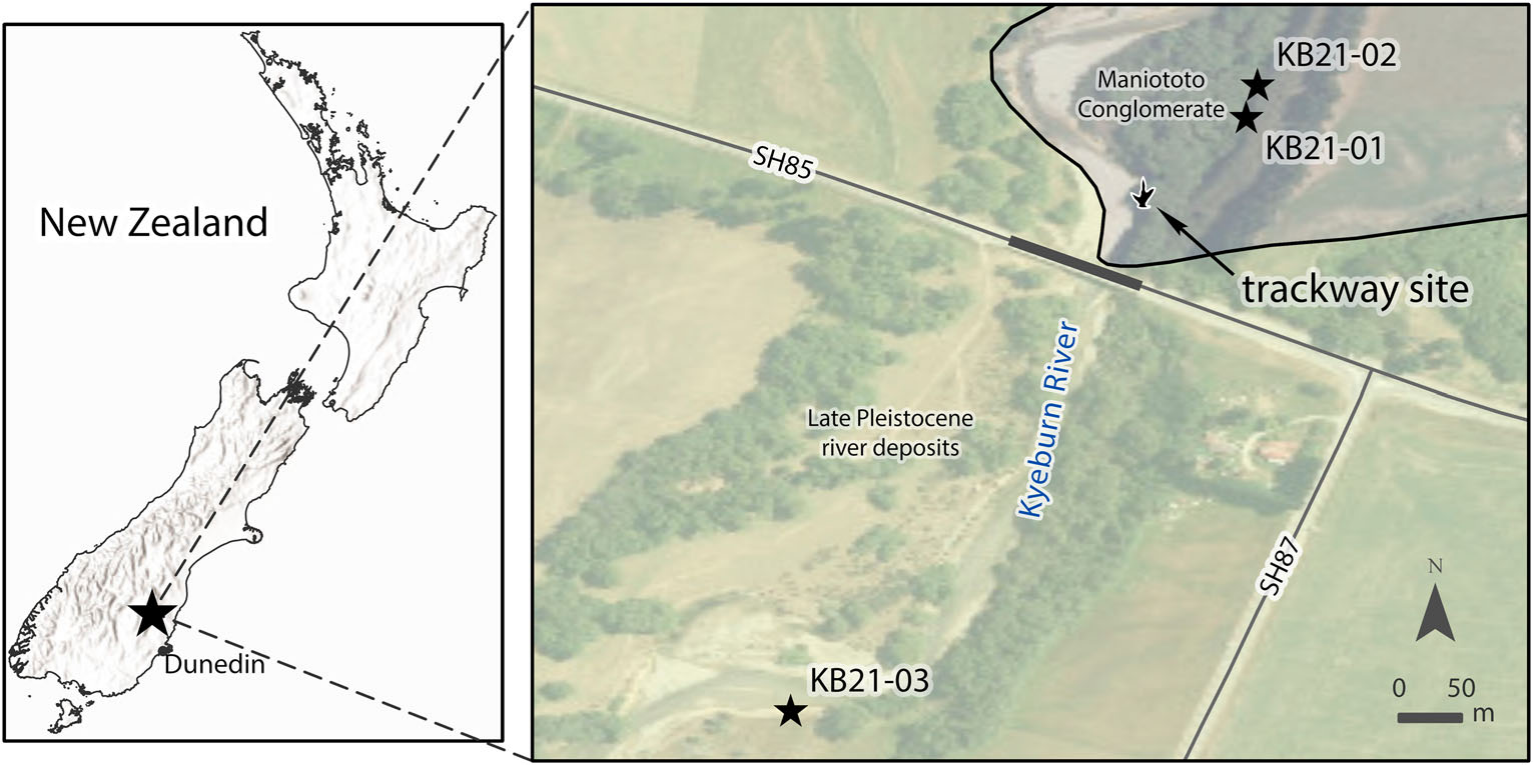
Map showing the location of the moa footprint trackway site and excavation site in relation to the Kyeburn Bridge that crosses the Kyeburn River on State Highway 85 in Central Otago, New Zealand. Temporary river diversion was ∼20 m to the west of the trackway site through already disturbed river gravels. Localities and field numbers of sediment samples extracted for cosmogenic nuclide dating, and extent of Maniototo Conglomerate outcrops are indicated.

**Figure 2.**
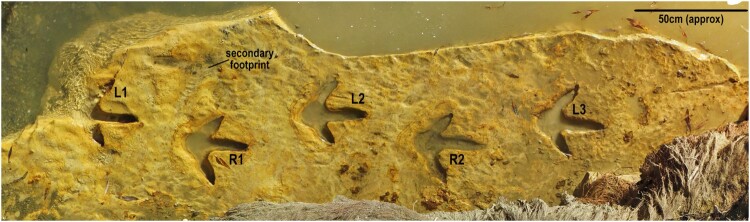
Orthographic image of the moa trackway found in the Kyeburn River immediately prior to extraction (OMNZ AV11896). Visible are five of the six footprints that were recovered as well as the very faint footprint discovered during the review of the orthographic imagery after the excavation and recovery. The larger shallow footprint can be seen between the first two footprints to the left. R3 and L4 are obscured from sight by the bank.

In March 2019, farmhand Michael Johnston alerted Tūhura Otago Museum to a trackway made up of tridactyl footprints exposed in the Kyeburn River upstream of the State Highway 85 bridge (−45.148442 ⁰S, 170.262257 ⁰E; WGS84) on the Maniototo plains of Central Otago (Figure 1). The imprints were preserved on a bedding surface of indurated silty clay on the riverbed and were likely exposed by a large flow event in November 2018. There were five visible prints in the trackway exposed in a line parallel to the riverbank (see Figure 1 and Figure 2) and another two that were partially obscured by the overhanging bank. A decision was made to extract the prints given the ephemeral nature of the site and the wish to preserve them for future generations. The footprints were under 1.3 m of water, so the entire river required diversion around the site to extract them. On 10 May 2019, after the appropriate resource consents and permissions were granted, a team consisting of members from: Tūhura Otago Museum, the University of Otago’s Department of Geology, Kāti Huirapa Rūnaka ki Puketeraki and Rūnaka o Ōtākou, excavated six of the footprints. Extraction was conducted using a masonry saw and chainsaw, with each footprint being removed individually.

### Registration and specimen information

**Figure 3.**
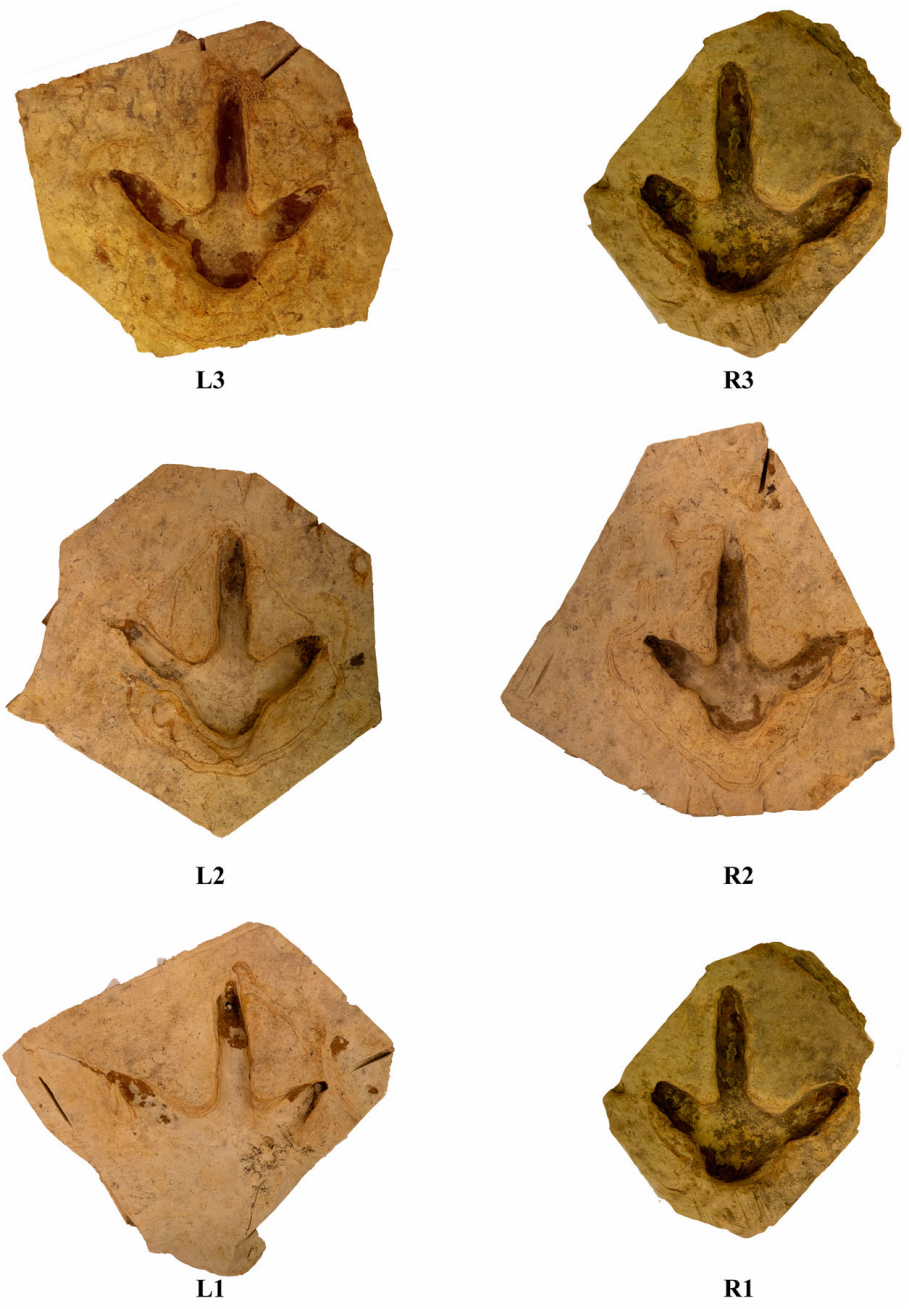
Side by side comparisons of the OMNZ AV11896 dried footprint blocks after the extraction from the Kyeburn River, Central Otago, New Zealand. Not to scale.

**Figure 4.**
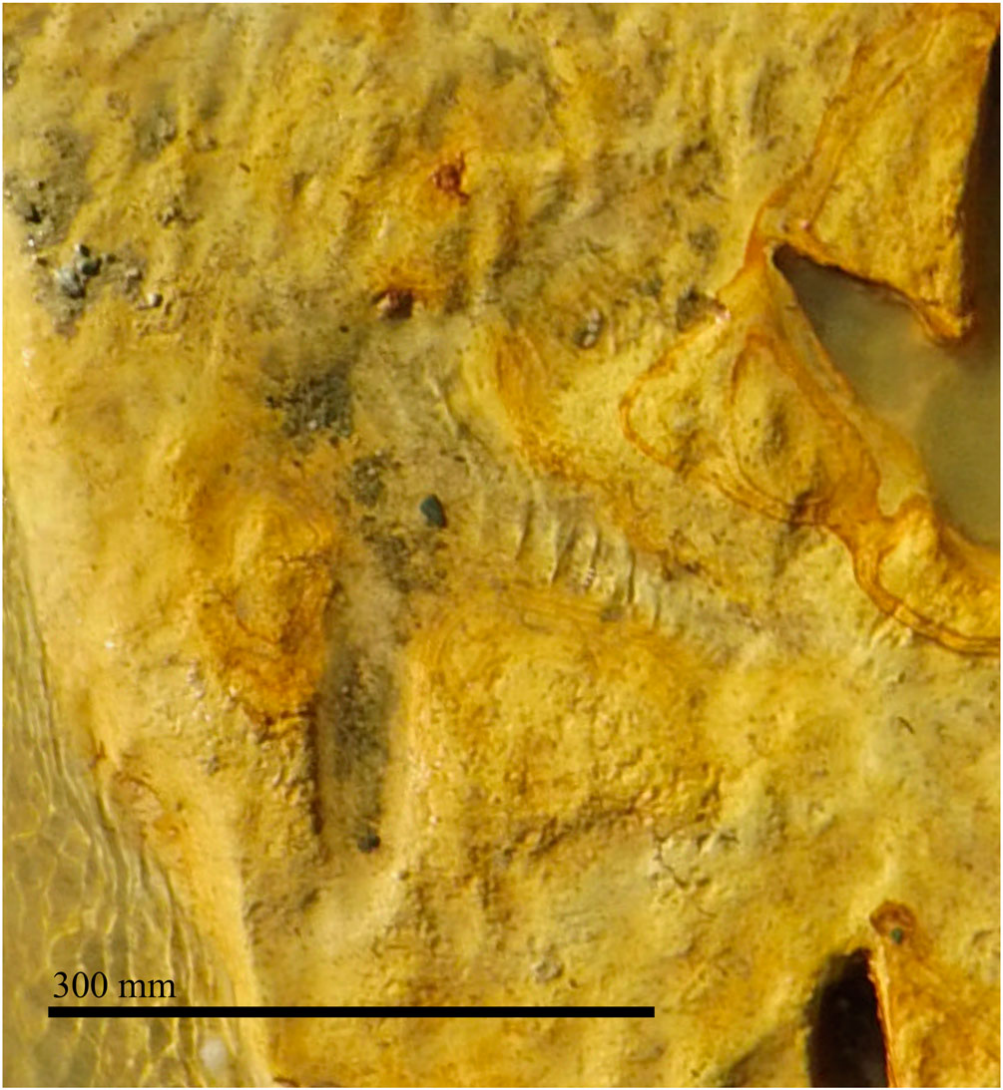
Close up of the associated larger footprint found in the digital imagery after the Kyeburn footprint excavation from Kyeburn, Central Otago, New Zealand.

The six footprints are lodged in the Tūhura Otago Museum, Dunedin (OMNZ AV11896). Locality and collection information is available on the New Zealand Fossil Record File Database (www.fred.org.nz) under fossil record number I42/f34. Subsequent inspection of the site survey imagery collected during the excavation captured the details of a larger moa footprint crossing the trackway (see Figure 2 between prints L1 and R1, footprints are labelled as per Figure 2. Larger photographs of the individual footprints can be seen in Figures 3 and 4).

### Geological setting

The moa trackway was found exposed on a bedding plane of buff-coloured indurated silty clay. The mudstone lithofacies can be traced upstream ∼200 m to where 30 m high cliffs on the true left side of the river valley meet the river’s bank. Here the riverbank outcrops are conformable with the Maniototo Conglomerate that comprises the cliffs (see Bishop, 1979). The Maniototo Conglomerate Formation (Youngson et al. 1998) is a series of indurated clays, sands and gravels dominated by greywacke clasts (Bishop 1974, pages 32–34; Bishop 1979, pages 13–16). These sediments represent a fluvial plain deposited by braided river systems that drained the newly uprising greywacke mountains of the Kakanui, Ida and Hawkdun ranges. These ranges were uplifting along the Waihemo-Hawkdun Fault Zone, which bounds the northern and north-eastern margins of the Maniototo Basin (Youngson et al. 1998; Forsyth 2001; Craw et al. 2016). Imbrication of Maniototo Conglomerate clasts at nearby localities indicates flow from the northeast indicating an emerging Kakanui range as the source (Bishop 1974). Youngson et al. (1998) inferred the fine-grained massive beds in the formation are loess deposits; wind-blown sediments that accumulated on the river braid plain. We infer that moa walked across the poorly compacted, fine sediments on the ground as they navigated the river braid bars and banks of the braid complex, leaving deep, clear pedal impressions. Further aeolian deposition rapidly buried the tracks, preserving them in high detail. Subtle differences in cohesion and/or grain size allowed the overlying horizon to be differentially eroded from the paleosurface by the recent flooding of the Kyeburn River. Liesegang rings surround the footprint depressions (Figure 2, 3 and 4), indicating that during diagenesis groundwater pooled on this irregular contact within the rock sequence, and resulted in the iron oxidation concentrations around the footprints. This extra lithification preserved the impressions’ surface morphology, resulting in the trackway withstanding the fluvial erosion that recently exposed the horizon. Note that print L1 was badly eroded by abrasion from the gravel bedload in the river during high water flow (Figures 2 and 3) and L4 was heavily damaged from overlying vegetation that was growing through the overlying sediment layers (not included in imagery or excavated).

### Geological age – previous work

There are few biochronostratigraphic constraints for the Maniototo Conglomerate, which is stratigraphically thick (thickness over 450 m in some places; Bishop 1974) and is likely diachronous across Central Otago. Fossil pollen was not recovered from samples analysed from the moa trackway horizon (E. Crouch, GNS Science, pers. comm., 2019) and adjacent outcrops (New Zealand Fossil Record file numbers I41/f8, f9); all samples appeared to be too weathered to preserve fossil pollen. Fossil pollen dating from blue-grey outcrops ∼15 km north in the upper Kyeburn River (New Zealand Fossil Record file number I41/f8596) yielded an age of the New Zealand Stage Opoitian or younger (Pliocene to present; Bishop 1974). These outcrops were interpreted by Youngson et al. (1998) to be the unweathered Maniototo Conglomerate. The formation was given a ∼11 Ma age range of Late Miocene to upper Pleistocene by Bishop (1974, 1979) and Youngson et al. (1998), though a Pliocene age was suggested by Forsyth (2001) and Craw et al. (2016, 2022).

## Materials and methods

### Nomenclature

Where possible, local Kai Tahu Māori placenames are given first in their spelling as per Māori pronunciation followed by anglicised spelling or the English name. Geological nomenclature that uses Māori placenames follows spelling from previous scientific publications, where any anglicised spelling is retained here for continuity in the literature.

### Geological dating

Minimum and best-fit ages were determined from the overlying sand and gravel sequences using cosmogenic nuclide burial dating (see Granger and Muzikar 2001 for a summary of the method). Cosmogenic nuclides are produced in near-surface rocks and sediments when high-energy cosmic radiation interacts with nuclei in rocks and minerals – in this case producing the radioactive isotopes ^26^Al and ^10^Be from oxygen in quartz. If the quartz is within the upper few metres of the surface, these nuclides will build up in the minerals (Dunai 2010). However, subsequent burial blocks the incident cosmic radiation with the result that the concentration of each isotope will reduce based on its half-life, 1.39 Ma for ^10^Be (Chemeleff et al. 2010) and 700 ka for ^26^Al (Norris et al. 1983). This technique has been used in the past to date alluvial sequences and moraines, including those containing early human artefacts and remains (see review by Granger 2006).

We collected two samples from a 30 m high cliff face (KB21-01 & KB21-02) ∼12 m stratigraphically above the clay layer containing the tracks. Sample KB21-01 (442069E, 5000353S, UTM Zone 59G) was 20.4 m below the top of the cliff and consisted of ∼12 well-rounded quartz pebbles, up to 2 cm in diameter, weighing a total of ∼200 g. Sample KB21-02 (442073E, 5000375S) was 22 m below the top of the cliff and consisted of ∼1 kg of fine to medium, well-rounded sand. A third sample, KB21-03 (441814E, 4999866S), was taken ∼100 m downstream of the tracks on the modern riverbank. The sample consisted of fine-grained sand. The samples were processed using standard procedures (Kohl and Nishiizumi 1992). They were crushed and sieved to 0.25–0.5 mm, magnetically separated and subsequently treated with HCl and HF to purify the quartz and remove any meteoric components. Quartz dissolution and extraction of the Be and Al fraction was carried out at the Australian Nuclear Science and Technology Organisation’s (ANSTO) cosmogenic geochemistry laboratory using ∼300 µg of ^9^Be from a low-level beryl carrier solution added prior to complete HF dissolution. Accelerator mass spectrometry (AMS) measurements of Be were performed at the 6MV SIRIUS accelerator facility (Wilcken et al. 2017, 2019). Results for ^10^Be were normalised to KN-5-3 with a reference value of 6.320e-12 (Nishiizumi et al. 2007) and ^26^Al analysis were normalised to KN-4-2 with a reference value of 3.096e-11 (Nishiizumi et al. 2004).

To determine the most-likely burial age, we constructed a model to track nuclide concentrations through time for different burial depths and durations (see supplemental file 1 for model details). The model accounts for nuclide production at depth due to neutrons and fast and stopped muons following Schaller et al. (2002), and decay based on the nuclide half-lives. The initial ^26^Al and ^10^Be concentrations were calculated assuming background erosion rates of 0.001–0.01 cm/yr. We allowed initial burial depths to vary from a maximum of 150 m to a minimum of the current depth of the sample. The time of initial burial was allowed to vary from 8 to 0 Ma. Finally, the samples are ‘exhumed’ at any time between the initial burial and today. The model sampled uniformly from these parameters 500,000 times. Goodness of fit was calculated as the sum of the absolute error between the modelled and measured ^26^Al and ^10^Be concentrations (i.e. a 1-sigma error indicates that the modelled concentration was within the 1-sigma error of the measured concentration). We modelled each sample independently using their respective concentrations and current depths.

### Trackway measurements

**Figure 5.**
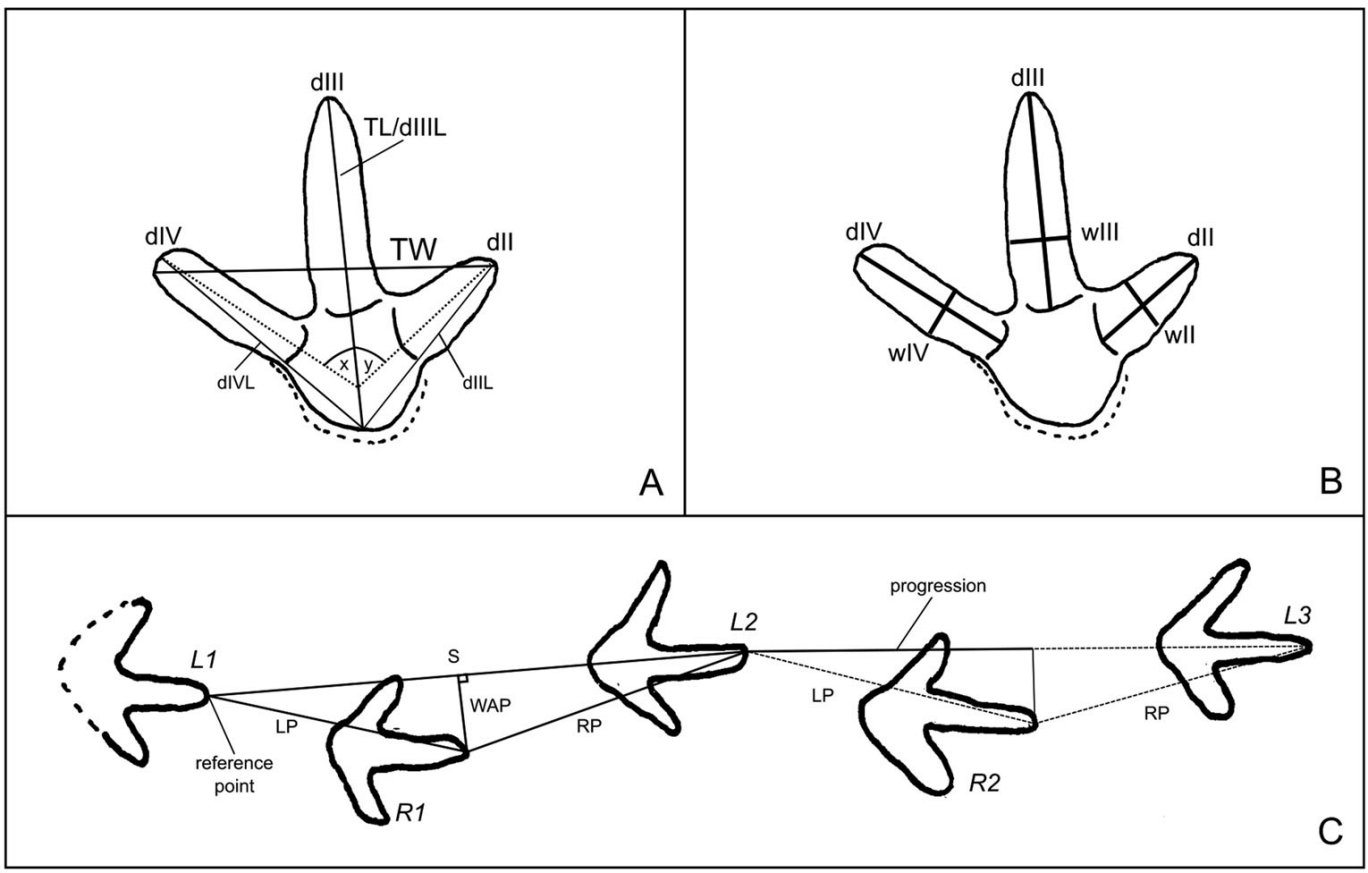
Methodology of footprint and trackway labelling and parameter measurements. The footprints represented in A and B are of a left foot. **A,** Total footprint length as measured from the tip of the claw of digit III to the caudal edge of the heel imprint (TL) and total width which is measured as the widest measurement from the end of digit II to III (TW). Total digit length is from the front of the claw or furthest end point of the digit impression to the centre of the caudal end of the heel imprint. In the schematic drawing, there is a faint external track outline around the heel pad imprint. The heel measurement was taken from within the footprint proper. Digit divarication between digits II-III (y) and III-IV (x) were taken from the mid-line of the digits and measured in degrees. **B,** Digit lengths from the tip of the claw to the rise between pad impressions (d) and widths at the thickest point of the digit (w). **C,** Trackway parameters. Labelling of the trackway starts with L1. LP and RP are the left and right paces respectively. S is the stride length. Width of the angulation pattern (WAP) which is measured perpendicular to the stride length. Progression for completely regular trackways would be half the stride length. The reference point used for the trackway parameter measurements is the tip of the third digit. Please note that this is a stylised schematic and is not drawn to scale.

Using photogrammetry, we created an orthographic model of the site before the excavation of the footprints. The model was calibrated against actual measurements of the footprints as well as a scale in the images to ensure its accuracy. Trackway descriptor measurements include stride length, step progression, and the width of the angulation pattern (WAP) as well as the pace angulation as per Bishop et al. (2017). The WAP was calculated using the cosine rule and trigonometry from the stride length and the step progression length. Figure 5 shows trackway measurement parameters and labelling. The numbering of the footprints is in order of occurrence from the first left footprint, this being L1 and the first right footprint being R1. Because of the complexity of working at the bottom of a riverbed of a large flowing river with a trackway that extended into a bank, not all footprints were able to be captured in the digital image. For footprints numbered R3 and L4 the trackway measurements were taken manually prior to removal from the riverbed.

### Footprint measurements

The following measurements from the trackway footprints were taken in millimetres: length of digit II, III and IV from the tip of the claw to the rise of the last pad impression of the digit (digit I was not possible to measure as it is the phalange that points backwards and it is not preserved in many moa). This rise is obvious on inspection of the footprints and formed an obvious landmark and can be seen in Figures 2, 3 and 5. Digit width at the thickest point which was consistently located approximately two-thirds down the digit (see Figures 2, 3 and 5). Total length of each digit from the claw tip to the most caudal part of the heel impression for digits II, III and IV. Total length (note, this measurement is the same as the total length for digit III but separate refence is useful for interpreting other footprints) and total width of the footprint measured as the widest possible measurement from within the footprint impression. Divarication angle between digits II-III, III-IV and II-IV was taken as the angle between the midlines of the digits. Measurements taken are shown in Figure 5. Maximum depth of footprint impression at the deepest point. All measurements were taken from the physical footprints themselves before they were dried. For the last footprint in the trackway (L4), the only measurement that was practicable was the depth of the print because of its location within the riverbank. For this reason, it was excluded in comparisons. To measure the single footprint made by a secondary print maker we used a calibrated orthographic model rather than the physical footprint.

### Hip height and speed of travel estimation

The trackmaker hip height was estimated by the formulae developed by Alexander (1976). The calculation for hip height (acetabulum height [h]) for bipedal track makers is;

h=4×footprintlength

As there are multiple footprints, an average length of the footprints was used.

Hip height estimates for the track maker was used in conjunction with Alexander's (1976) equation where SL = stride, h = hip height, g = acceleration of free fall;

$\mathrm{speed}\approx 0.25\mathrm{speed}\;≅0.25\;\surd g\times SL^{1.67}\times h^{-1.67}≅0.7826\times SL^{1.67}\times h^{-1.17}$

### Mass of the Kyeburn moa

We have predicted the mass of both moa individuals represented as tracks based upon the allometric linear regression models developed by Alexander (1983) which is in line with other estimates of moa by Duncan and Holdaway (1989). We used the linear regression equation and constants from Alexander (1983) to predict the weight based on the longest digit with the equations and values as follows;

$$y/y_{o}=(m/m_{o}{)}^{b}$$

where *y* is the length of the longest digit in mm, *m* is the body mass in kg, *y_o_* (178), *m_o_* (92), are geometric mean values calculated by Alexander (1983) for Anomalopterygidae (synonym of Emeidae, see Checklist Committee 2022) for toe length. *b* (0.26) is used from the Alexander (1983) Model I. For the trackway maker, the results presented represent the mean length of digit III with the maximum and minimum values based on the length of the same digit from the footprints. Digit length is used because the equations produced by Alexander (1983) were derived for skeletal material rather than footprints. For the other larger footprint, we present the weight as a single value rather than a range as we only had one footprint to measure digit III from.

### Comparative ichnology

The measurements of the footprints were compared to complete articulated moa foot skeletons from the collections of Tūhura – Otago Museum, Southland Museum and Art Gallery and Waitaki Museum. Only moa foot skeletons that were complete enough to get most of the measurements were used. We measured individuals from the species of moa that were from lowland South Island taxa according to Bunce et al. (2009). These are little bush moa (*Anomalopteryx didiformis*), stout legged moa (*Euryapteryx curtus gravis*), eastern moa (*Emeus crassus*)*,* heavy-footed moa (*Pachyornis elephantopus*) and South Island Giant Moa (*Dinornis robustus*)*.* Due to sexual dimorphism, we have treated male and female *D. robusus* separately. We have also included in this analysis upland moa (*Megalapteryx didinis*) as, although unlikely, there is a possibility that the Pliocene-Early Pleistocene habitat of the Maniototo could have overlapped with the Holocene habitats known for this species. From these moa skeletons, we measured the length of digits (sum of phalanges including the claw), width at the thickest point of the digit, as per Figure 5B (all specimen registration numbers and raw data are presented in supplementary file 2).

Please note that due to the comparison that we are making here between the footprints and skeletal material we have not performed any statistical comparisons and have drawn our conclusions from the variation and observations alone, we have not done other measurements due to the historical inaccuracy of mounted moa skeletons, for example the angle of the phalanges and the digits lacking cartilage. When interpreting results, it should be noted that mummified remains show that the thickness of flesh on each side of the digits and areas of the foot lacking fat pads can be up to 5 mm thick in the smallest moa, *A. didiformis* (K. Fleury pers. obs. 2020 – mummified remains from Otago Museum (OMNZ AV7474) and Southland Museum (E80.4)).

## Results

### Geological age

**Figure 6.**
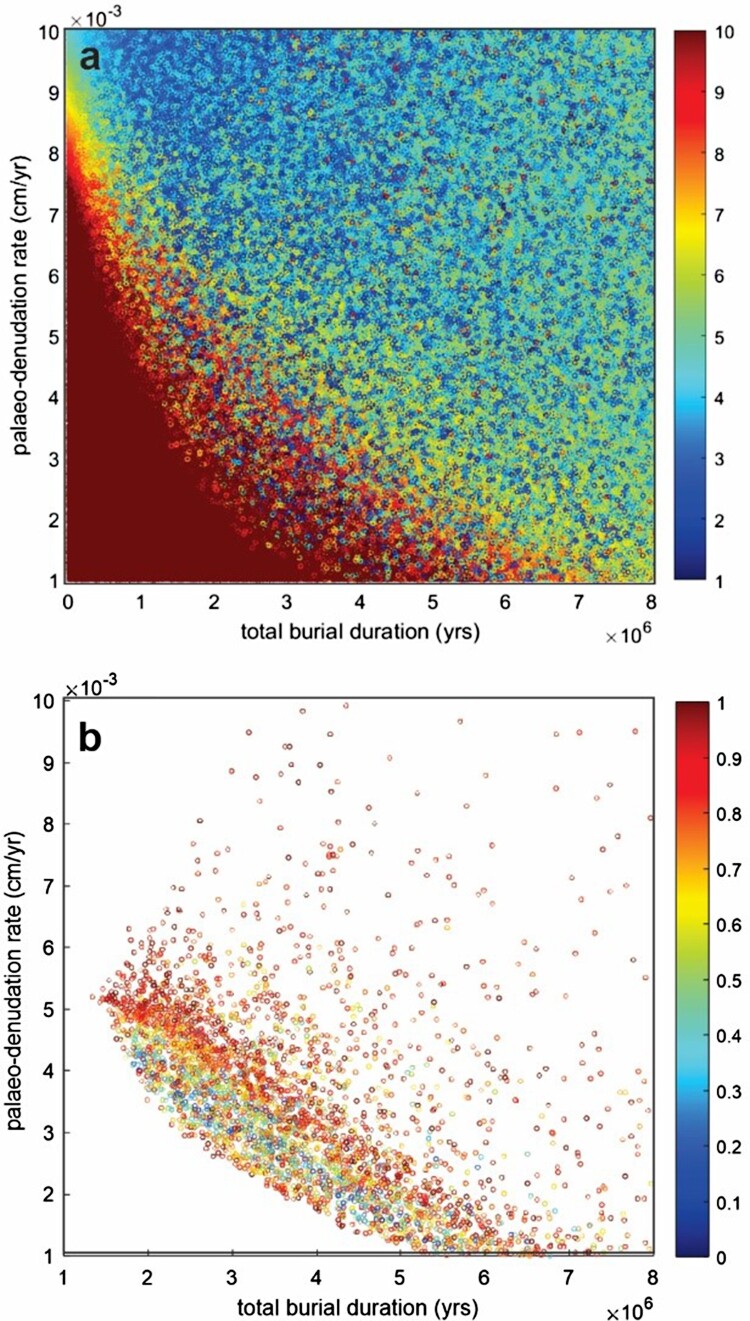
Results of the Monte Carlo burial model for KB21-01. The top panel (a) includes all 500,000 model results, demonstrating the overall poor fits for younger ages. The colour scale is the goodness of fit as measured by standard deviation from the measured concentrations. Panel (b) shows only those results that were within 1-sigma of the measured values. Note the covariance between paleo-denudation rates and modelled ages.

**Figure 7.**
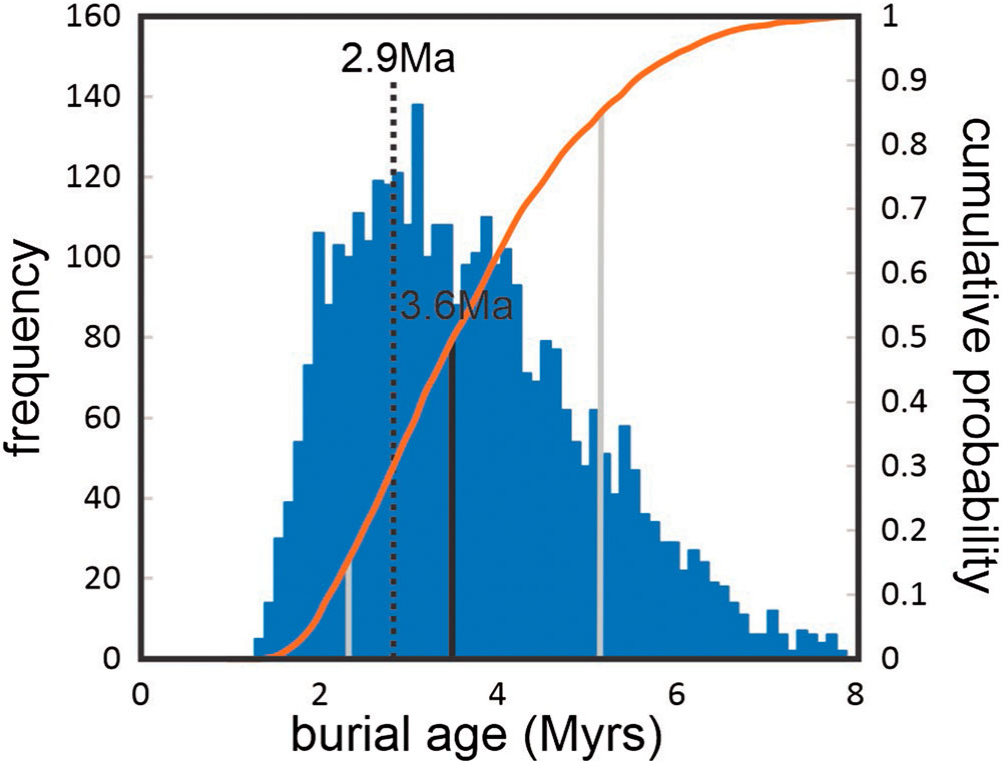
Frequency and cumulative probability of best fit modelled ages from sediment samples KB21-01 & KB21-02. The mean of the distribution is 3.57 Ma (+1.62/−1.18 at 1-sigma). The distribution is skewed with a mode of 2.9 Ma and a long tail towards older ages.

Samples KB21-01 and KB21-02 resulted in indistinguishable outputs and are both different from KB21-03 which is part of a different sedimentary complex (Table 1). Of the 500,000 model runs, over 3000 matched the measured concentrations within 1-sigma (Figure 6). The model results demonstrate the covariance between the palaeo-denudation rate (the erosion rate of the catchment that supplied the quartz) and the modelled burial age. Faster erosion delivers lower ^26^Al and ^10^Be concentrations, requiring shorter burial durations to decay down to the measured concentrations. Note, however, that there is a limit to this relationship (Figure 7) with palaeo-denudation rates faster than 0.005 cm/yr yielding fewer plausible burial scenarios. While low for tectonically active regions, this corresponds well with the global mean denudation rate from modern catchments (Portenga and Bierman 2011). The model results also suggest that the samples were initially buried to a depth of 74 (+44/−34) m and that exhumation to 2040 cm below the surface occurred at 410 (+330/−280) ka, however, the wide distribution of the burial depth and exhumation time limits their usefulness. The burial age, and hence the minimum age for the moa tracks is better constrained with a mean of 3.57 Ma (+1.62/−1.18) with a mode of 2.9 Ma. No acceptable models for KB21-01 and KB21-02 resulted in ages younger than 1.4 Ma (Table 1).

**Table 1.** Accelerator mass spectrometry results from cosmogenic nuclide burial dating using Al and Be.

**Sample Name**	**Sample mass (g)**	**^26^Al/^27^Al**	**σ(^26^Al/^27^Al)**	**sigma [%]**	**blk correction [%]**	**Al [mg/kg]**	**σ (^27^Al) [mg/kg]**	**^27^Al [ atoms ]**	**^26^Al [ atoms ]**	**σ (^26^Al) [ atoms ]**	**^26^Al [ atoms/g Qtz ]**	**σ (^26^Al) [ atoms/g Qtz ]**	**σ (^26^Al) [ % ]**
KB01-Al	10.071	1.3206E-14	2.01822E-15	15.3	4.0	332.8	3.328	7.48E + 19	9.756E + 05	1.51E + 05	96877	15034	15.5
KB02-Al	15.046	1.842E-14	1.83313E-15	10.0	2.9	242.2	2.422	8.13E + 19	1.486E + 06	1.50E + 05	98750	9967	10.1
KB03-Al	20.038	2.389E-14	1.76327E-15	7.4	2.2	89.5	0.895	4.00E + 19	9.440E + 05	7.15E + 04	47112	3570	7.6
Al_Blk		5.2835E-16	3.05089E-16	57.7									
**Sample Name**	**Sample mass (g)**	**^10^Be/^9^Be**	**σ(^10^Be/^9^Be)**	**sigma [%]**	**blk correction [%]**	**^9^Be [ug]**		**^9^Be [atoms]**	**^10^Be [atoms]**	**σ (^10^Be) [atoms]**	**^10^Be [atoms/g Qtz]**	**σ (^10^Be) [atoms/g Qtz]**	**σ (^10^Be) [%]**
KB01	10.071	1.881E-14	1.03E-15	5.5	14.3	265.00		1.770E + 19	3.887E + 05	1.84E + 04	3.306E + 04	1.82E + 03	5.5
KB02	15.046	2.922E-14	1.31E-15	4.5	9.7	263.83		1.762E + 19	5.704E + 05	2.35E + 04	3.422E + 04	1.56E + 03	4.6
KB03	20.038	1.407E-14	9.01E-16	6.4	18.3	265.24		1.772E + 19	3.051E + 05	1.61E + 04	1.244E + 04	8.03E + 02	6.5
Blank		3.150E-15	5.41E-16	17.2									

KB23-01 and KB23-02 are part of the Maniototo Conglomerate formation in the outcrops above the state highway 85 bridge and KB23-03 is part of the much younger downstream Kakanui range protolith that is below the bridge.

### Trackway description

The trackway featured seven large tridactyl footprints that were on average 46.3 mm deep (sd = 5.8) and were between 272 and 300 mm wide and between 194 and 260 mm in length that were made by a single individual (detailed measurements are presented in Tables 2 and 3). The average divarication angle in degrees between digits II-III was 48.50 (sd = 2.4) and III-IV 54.17 (sd = 3.4).

**Table 2.** Detailed Kyeburn footprint measurements taken from each individual footprint in the trackway and of the second individual footprint made by another moa.

	Total Length	Total Width	Digit II length	Digit III length	Digit IV length	Width of Digit II	Width of Digit III	Width of Digit IV	II-III Digit Divarication angle	III-IV Digit Divarication angle	Depth	Digit II claw to heel	Digit III claw to heel	Digit IV claw to heel
L1*	250	294	95	170	115	39	40	38	49	51.5	37	210	250	210
R1	260	295	110	161	112	50	43	53	45	58	51	199	260	205
L2	278	272	99	175	120	48	40	34	52	51	44	177	278	217
R2	294	296	105	190	124	41	45	40	50	56	44	192	294	215
L3	281	300	109	179	137	49	46	42	47	51	50	186	281	223
R3**	280	282	105	170	119	45	44	44	48	57.5	52	185	280	210
L4***											30			
Secondary foot print maker ****	285	448	170	205	168	66	58	55	60.3	60.3	na	266	285	283

Measurements were taken before the drying process began except for the secondary footprint, measurements were taken from imagery collected during the excavation. Of these footprints, only L1, R1, L2, R2, L3 and R3 were collected and now reside at Otago Museum as OMNZ AV11896. For the secondary footprint, all measurements were collected from orthographic imagery constructed after the excavation.

* Print badly eroded, measurements estimate only, depth is of the middle toe due to eroded state; ** Digit II distal end extends 21 mm into the block, the measurement of digit length includes this; *** Print not collected from river. Badly eroded from plant roots that were growing into and around the impression. Print hidden under bank; ****Print not collected from the river and all measurements were taken from imagery.

**Table 3.** Kyeburn moa trackway stride length, progression length and walking angular progression (also known as gauge) measured before excavation from within the river and from the calibrated orthographic model of the trackway.

	Stride length (mm)	Notes
L1-L2	989	
R1-R2	1020	
L2-L3	1030	
R2-R3	980	Measured in the riverbed
L3-L4	1070	Measured in the riverbed
	**Progression length (mm)**	
LP1	491	
RP1	537	
LP2	535	
RP2	526	
LP3	480	Measured in the riverbed
	**Walking angular progression**	
	WAP (mm)	
L1-R1-L2	140.055	
R1-L2-R2	164.9114	
L2-R2-L3	127.2946	

The sequence of the footprints shows the track-making moa was travelling in a north-north-east direction. The trackway sequence was greater than 2 m long and 0.4 m wide. The posterior section of the first footprint in the series (L1) shows evidence of substantial erosion due to it being covered in shingle in the river. The average sizes of the footprints in the sequence are presented in Table 4.

**Table 4.** Average sizes of the Kyeburn footprints and those measured from skeletal elements from Tūhura Otago Museum, Waitaki Museum and Southland Museum collections.

	digit II		digit III		digit IV	
	Length	Width	Length	Width	Length	Width
**Kyeburn Trackmaker**	103.8 (sd = 5.8, *n* = 6)	45.3 (sd = 4.5, *n* = 6)	174.2 (sd = 9.8, *n* = 6)	43 (sd = 2.5, *n* = 6)	121.2 (sd = 8.8, *n* = 6)	41.8 (sd = 6.5, *n* = 6)
**Secondary Footprint**	170 (*n* = 1)	66 (*n* = 1)	205 (*n* = 1)	58 (*n* = 1)	168 (*n* = 1)	55 (*n* = 1)
**Emeidae**						
*A. didiformis*	118 (sd = 25, *n* = 3)	23.2 (sd = 1.8, *n* = 3)	145.5 (*n* = 2)	25 (sd = 1.3, *n* = 3)	142 (*n* = 2)	22.5 (*n* = 2)
*E. crassus*	109.2 (sd = 9.4, *n* = 5)	23.7 (sd = 3.4, *n* = 5)	146.75 (sd = 1.7, *n* = 4)	26.9 (sd = 3.2, *n* = 5)	115 (sd = 17, *n* = 5)	24.6 (sd = 2.7, *n* = 5)
*E. curtus*	119.3 (sd = 5.9, *n* = 3)	29.8 (sd = 2.8, *n* = 3)	150 (sd = 6.2, *n* = 3)	32.7 (sd = 1.2, *n* = 3)	115.7 (*n* = 2)	27 (sd = 1, *n* = 3)
*P. elephantopus*	140 (sd = 10.7, *n* = 7)	34.9 (sd = 4, *n* = 8)	181.3 (sd = 20.1, *n* = 8)	39.6 (sd = 6.2, *n* = 8)	131.1 (sd = 17.6, *n* = 7)	33.5 (sd = 3.2, *n* = 8)
**Dinornithidae**						
*D. robustus. (M)*	156.5 (sd = 19.2, *n* = 3)	33 (sd = 2.7, *n* = 3)	195.7 (sd = 33.3, *n* = 3)	36.2 (sd = 4, *n* = 3)	163.3 (sd = 9, *n* = 3)	3 (sd = 4.4, *n* = 3)
*D. robustus. (F)*	193 (sd = 15.1, *n* = 6)	41.3 (sd = 0.8, *n* = 6)	240.3 (sd = 18.3, *n* = 6)	43.9 (sd = 0.8. *n* = 6)	211 (sd = 11.7, *n* = 6)	39.83 (sd = 1.1, *n* = 6)
**Megalapterygidae**						
*M. didinus*	137 (*n* = 1)	22.5 (*n* = 1)	176 (*n* = 1)	26 (*n* = 1)	*n*a	23.5 (*n* = 1)

Lengths are the mean lengths and widths measured in millimetres of the moa digits with standard deviations and sample size for each measurement are presented in brackets. Specimen registration number and raw data are presented in supplementary file 2.

The stride length between the first left footprint and the second left footprint, L1 and L2, was 989 mm long, between R1 and R2 was 1020 mm and between L2 and L3 1030 mm. The physically measured stride length between R2 and R3 was 980 mm and between L3 and L4 was 1070 mm (please note that there were two more footprints that were obscured in from the orthographic image). Overall, there was a small direction change travelling to the right. The walking angular progression and the stride length are presented in Table 3. The WAP or gauge between L1-R1-L2 is 1450.05 mm, R1-L2-R2 is 164.91 mm and L2-R2-L3 is 127.29 mm showing how far each footprint extends out from the midline of progression.

### Footprint descriptions

There was no impression for Digit I, the hallux, visible in or near any of the footprints. Digit II is shorter and thicker than digit IV, this and the following descriptive features can be seen in Figure 3. Digit III is longer than II and IV and is directed forwards in the line of travel. Some of the footprints have what look to be claw impressions at the distal end of the phalanges where the impression terminates. The best examples of this are seen in footprint R1 and L3 where the claw marks are obvious for digits II and IV and appear rotated outwards. The impression for the ungual of digit III is rounded, lacking a narrowing pointed tip except for what is perhaps a faint mark at the distal end of footprint R2. The impression left by the fleshy metatarsal pad is asymmetrical with a shorter steeper inside margin than the outside one and well pronounced in all footprints except for the damaged footprint L1. Figure 3 shows enlarged views of all of the six footprints that were collected.

The plantar surface of the moa foot consists of fleshy integument that covers and protects the joints between the phalanges. There is a single undivided impression for the plantar pad of digit II in all the footprints. On digit III, there are two parts to the impression suggesting a divided fleshy pad evidenced by a faint widening of the wall and a discernible interpad space. We assume that there was a third pad that connected the distal phalanges to the claw, but it is not obvious. The impression at the distal end of digit III has collapsed around the claw which obscures the ability to clearly discern the interpad space and the claw impression of this digit. Of all the digits this is the hardest to discern specific details. Digit IV shows impressions for two fleshy digital pads and this is the clearest in footprints R1, L2 and L3.

The variations in length of the footprints are caused either by a collapse in the sediment, erosion within the river, or the growth of an iron-rich layer within the footprints which is harder than the surrounding sediment. Footprints R1 and L2 show the best preservation. No striations or other marks or skin textures were able to be discerned by the authors. There is in several of the footprints, a hollow under the surface where the claw was pushed into the mud. This indicates that during the stride as the moa lifted its foot, the digits were rotated plantar-posteriorly as the pressure of the foot was released, otherwise striations or drag marks should be visible. Each footprint has a very clear rounded metatarsal pad that has a defined mark that separates it from the digits. At the rear of each footprint is what looks to be a point where the metatarsal pad has rounded off the edge of the footprint as the foot was being placed onto the mud.

### Secondary footprint description

The larger singular footprint was travelling in a south-east direction. It was only observed in the orthographic images taken when it was covered in water as a highly eroded impression that was barely visible (see Figure 4 for an enlarged view). It was a tridactyl print that was at its greatest dimensions 285 mm long from the end of the metatarsal pad to the end of the impression made by digit II and 448 mm wide. This footprint was made by an individual that had a wide interdigital angle and longer digit length than the other trackway maker. There were no obvious digital joint pad impressions but the rounded area at the back of the foot where the metatarsal pad would have been seen. This footprint can be seen in Figure 2 and enlarged in Figure 4.

### Estimated Hip height and speed of travel

The estimated hip height of the moa that created the track way is 1095.33 mm, with an average stride length of 1018 mm. The average estimated speed of travel when the footprints were deposited is 2.61 kmh^−1^. The speed of the moa along the trackway did vary according to the stride length which at its minimum was 2.45 kmh^−1^ and at its maximum 2.81 kmh^−1^.

### Mass of the moa

We calculated that the moa that left the trackway had a mass of 84.61 kg with an estimated minimum of 62.53 kg and a maximum of 118.24 kg based on the mean length of digit III and its minimum and maximum measurements from the trackway. For the moa that left the second, larger footprint, the estimated mass is 158.375 kg.

### Species identification and comparison to articulated moa feet bones

#### Footprint morphometrics and comparisons to recent moa

Digit length comparisons shown in Table 4 demonstrate the differences between family and species-level groupings when comparing the sizes of the footprints to the bones of the feet. The moa responsible for the trackway had a relatively short digit II (mean = 103.8 mm; sd = 5.8 mm). This is most consistent with those species from the Emeidae family and was within the size range of that of *Emeus crassus* and *Anomalopteryx didiformis*. The lengths of digit III for the track maker (mean = 174.2 mm; sd = 9.8 mm) and digit IV (mean = 121.2 mm; sd = 8.8 mm) was most similar to the Emeid species *P. elephantopus* even though digit II was ∼35 mm shorter*.*

Width of digits of the trackway maker at the widest point (II: mean = 45.3 mm; sd = 4.5 mm, III: mean = 43 mm; sd = 2.5 mm, IV: 41.8 mm; sd = 6.5 mm) were all of a similar width to that of the Dinornithidae female *D. robustus* although there was considerable overlap with that of the male *D. robustus* and with the top end of the variation observed in the largest Emeidae species *P. elephantopus*. These are presented in Table 4. It should be noted that these comparisons are made between a footprint of a bird and with skeletal remains that have no flesh and the width of the bones with flesh on them would be ∼10 mm wider than the bone.

The length measurements of the bones also lack cartilage and other connective tissue.

Considering the combination of all the results here including length and width of the digits, it is our belief that the moa that left the footprints was most likely to be a large Emeidae individual with a high probability of it being related to the moa from the *Pachyornis* genera.

#### Secondary footprint species

The digits of the second footprint are of similar length to those of *D. robustus* (digit II: 170 mm, digit III: 205 mm, digit IV: 168 mm) and is most similar in proportions to that of a male *D. robustus*. Only digit IV is within the upper range of that measured of *P. elephantopus*. The widths of the print are larger than the widest female *D. robustus* at 66.1 mm, 58.3 and 55.2 mm for the respective digits II, III and IV. Because of the highly eroded nature of the footprint impression, it is likely that this print would have originally been larger than what was recorded. The print is most consistent with that of a species of *Dinornis*. We feel strongly that it is very similar to a female South Island giant moa because of the widths and lengths of the digits. The measurements of these are presented alongside that of the other footprints and moa species in Table 4.

## Discussion

The results of cosmogenic nuclide dating give best fit modelled ages for burial of the Maniototo Conglomerate at the Kyeburn River site with a mean age of 3.57 Ma (+1.62/−1.18 at 1-sigma) that is skewed with a mode of 2.9 Ma, and a burial age of no younger than ∼1.9 Ma (Supplementary File 1). An optimised age of Late Pliocene (Waipipian – Mangapanian; 3.7–2.4 Ma) encompassing the modelled best fit mean and mode is inferred by the authors to indicate the depositional age of the local strata of Maniototo Conglomerate when moa tracks were formed in the sediments. Encompassing the 1-sigma best fit modelled age range and the minimum acceptable burial age yields a more conservative age range of Pliocene to early Pleistocene (Opoitian – Nukumaruan; 5.33–1.63 Ma), though the refined Late Pliocene age range is adopted here. The footprints were found ∼12 m down sequence of the horizons of samples KB21-01 and KB21-02. The braided river system indicates a steep drainage that would be rapidly infilling the landscape as the local ranges uplifted, so the depositional age of the moa trackway horizon is unlikely to be appreciably older than the overlying dated beds on a geological timescale, The KB21-03 riverbed outcrops downstream of the State Highway 85 bridge are proposed to belong to younger deposits of a similar Kakanui range protolith (Figure 1; see Supplementary File 1), in part consisting of reworked Maniototo Conglomerate. Downstream of the Kyeburn River Bridge, Bishop (1979) mapped the riverbed stratigraphy as a younger stratigraphic unit and did not show Maniototo Conglomerate in or adjacent to the Kyeburn riverbed there.

The footprints were likely made by moa walking across a subaerially exposed part of the ancient braided river plain covered in loess, being buried rapidly by further glacial windblown dust (i.e. loess). The Late Pliocene depositional age can be used as a proxy for the minimum age for Neogene re-activation of the Waihemo-Hawkdun Fault Zone. This tectonic uplift provided the source of low-metamorphic grade protolith for the fluvial deposit; the Maniototo Conglomerate is the first sedimentary unit up sequence to be greywacke-dominated (Youngson et al. 1998; Craw et al. 2016, 2022). The Kyeburn River bridge locality particularly informs us about the timing of ‘unroofing’ of the basement rocks on the adjacent Kakanui mountain range which is the inferred river source from imbrication (Bishop 1974; Youngson et al. 1998).

It is possible to assign trackways to individual moa species similar in proportion to Holocene species (see Farlow et al. 2013), however, this requires caution especially when analysing >1 million year old tracks. Molecular divergence data from ancient DNA shows that the moa speciation event that resulted in the recently-extinct nine species occurred approximately ∼2 Ma and the moa families of Emeidae, Dinornithidae and Megalapterygidae diverged at ∼5 Ma (Bunce et al. 2009). The moa that made the trackway and accessory larger print likely lived in the Late Pliocene (Waipipian – Mangapanian; 3.7–2.4 Ma), which means that we can be highly confident of family-level identification and tentatively propose a genus-level identification based on recent taxa. It is important that we are cautious with the identification provided as we do not have skeletal material from moa from that period to compare with the footprints and there may be undocumented body size changes within moa lineages or even major extinction events in the last 5 Ma. Using our conservative Kyeburn moa footprint burial age of Pliocene to Early Pleistocene (Opoitian – Nukumaruan; 5.33–1.63 Ma), the Kyeburn footprints are the oldest footprints attributed to Dinornithiformes, and the oldest moa fossil remains to be attributable to family-level classification. Our refined burial age of Late Pliocene (Waipipian – Mangapanian; 3.7–2.4 Ma) indicates that the Kyeburn footprints are also the second oldest moa fossils of any form reported to date. Pre-Nukumaruan (Early Pleistocene; 2.4–1.63 Ma) records of moa are only known elsewhere from the lacustrine Bannockburn Formation of Early Miocene age (Altonian; 18.7–15.9 Ma) as a few bones and eggshell fragments of a least two different taxa, representing the earliest moa fossils yet found (Worthy et al. 1991; Tennyson et al. 2010). The footprints documented here are an important occurrence record in time and space for Dinornithiformes.

The hip height and mass estimates look to fit what we expect for an animal the size of one from the genus *Pachyornis* at 1095.33 mm and 84.6 kg (Alexander 1983b; Worthy and Holdaway 2002; Latham et al. 2020). These weight and height estimates make us believe that it was too big to be from the average to small individuals in genera *Euryapteryx* and *Emeus* but does leave it within the size of *Dinornis.* However, based on the dimensions we strongly believe it was more likely to be from the Emeidae family which rules out *Dinornis*. The speed of travel across the clay looks to be the equivalent of a slow walk at a speed of between 2.45 kmh^−1^ and 2.81kmh^−1^ across what would likely have been a slippery surface. These speeds are consistent with those estimated by Duncan and Holdaway (1989) from the Hill (1895) report of footprints from Tūranganui, Gisborne and is slower than the trackways from the Manawatū River.

The step width as measured by the walking angular progress (WAP) in this study of these footprints shows that this bird was travelling slowly across the silty-clay surface of which it left the footprints. Generally, in large bipedal animals like those of non-avian theropods, emu (*Dromaius novaehollandiae*), ostriches (*Struthio camelus*) and people, WAP decreases with speed (Bishop et al. 2017). For *Euryapteryx*, Worthy (1989) used femur shape to predict that it had a wider WAP than that of *Pachyornis*, showing that this can also vary between species. In taller bipedal animals the step width is very closely tied to mediolateral stability during bipedal locomotion and there is likely to be a trade-off between the efficiency of walking and forward movement (Bishop et al. 2017). This short trackway did not show any changes in gait or relevant changes in speed, so the step width was relatively constant but adds weight to the accuracy of the calculations that the moa was moving slowly across the substrate. Foot-morphology and function in birds is diverse and adaptations to assist in control of the foot-substrate interactions are important in maintaining balance, movement and injury avoidance in the environment.

There is a consistent pattern of placement and withdrawal in the three main toes of the trackway. Studies of other tridactyl species show that during sinking and withdrawal in soft substrates, the sub-surface path of the central toe (digit III) forms a consistent loop, where after foot placement and during the withdrawal of the foot from the soft surface there is a negative arc like motion where the digit moves backwards and exits behind the entry point. During the placement and sinking phase of digit II and digit IV they both spread until they reach the maximum depth in the substrate. As the foot is withdrawn from the substrate the side toes collapse and draw towards the central toe as it is lifted out of the substrate (Turner et al. 2020). The outward directed claws on digits II and IV support this and may provide some explanation as to some of the claw imprints of some of the prints extending into the mud beyond what is visible from the surface.

The use of modern imaging techniques before the footprints were removed was critical in the discovery of the second large footprint. The trackmaker in this instance, was most likely from the Dinornithidae and the size of the footprint was very close to that of a modern female *Dinornis*. This footprint appears at least to be of similar shape, digit spread and size to the other footprints that have been attributed to recent Dinornithidae from Palmerston North. It is not consistent with the smaller morphotypes from Gisborne which adds confidence to our identification (Hill, 1894; Lockley et al. 2007; Williams 1872). Our mass calculations have this moa at 158.375 kg which is in line with a female *Dinornis* (Worthy and Holdaway 2002; Latham et al. 2020). The discovery of this footprint in the imagery highlights the importance of proper site documentation before extractions occur. Because of this footprint having a very shallow impression and the level of erosion that had occurred, it was nearly lost without record.

Previously, moa footprint trackmakers in the North Island of New Zealand have been attributed to several different morphotypes by Lockley et al. (2007). These can, with a high degree of confidence be attributed to Dinornithidae and Emeidae. Three of these footprint makers were comfortably attributed to *Dinornis novaezealandiae* and one to each of *Euryapteryx curtus, Anomalopteryx didiformis* and *Pachyornis geranoides* by Lockley et al. (2007), though it should be noted that these were from younger deposits than the Maniototo Conglomerate tracks described here. This study adds two more morphotypes to the documented trackway marker list with South Island Dinornithidae and Emeidae and adds to the fossil record of moa for an era where few specimens available.

The single large footprint from the Kyeburn river locality provides evidence of moa attaining a body size comparable with Holocene *Dinornis*. Tennyson et al. (2010) reported Dinornithiformes eggshell from the early Miocene of the Bannockburn Formation, with the larger shell fragments similar to those of *Pachyornis* to *Dinornis* in size indicating a possible body mass range of 34–242 kg. Whilst those specimens are significantly older than the Kyeburn specimen, the greater ambiguity of body size determination from the egg shell fragments makes the larger Kyeburn footprint the earliest definitive record of moa reaching the gigantic *Dinornis* size class.

The discovery and description of the South Island’s first moa trackway is a major step towards understanding and appreciating Cenozoic Zealandia’s largest herbivores.

## Supplementary Material

Supplemental File 1

Supplemental File 2 data
