# Supplementary material for: The moa footprints from the Pliocene – early Pleistocene of Kyeburn, Otago, New Zealand
Source: J R Soc N Z. 2023 Nov 14;54(5):620–42. doi: 10.1080/03036758.2023.2264789 (PMC11459810; doi:10.1080/03036758.2023.2264789)
Supplement: Supplemental File 1 [file TNZR_A_2264789_SM6179.docx]

Supplemental file 1: Details of cosmogenic nuclide sampling and modelling approach.

Three samples of sand to gravel size quartz-bearing material were collected for cosmogenic nuclide burial dating on the 10^th^ of December, 2021 (Figure S1). Two samples (KB01 and KB02) were from stratigraphically above the moa tracks (Figures S2, S3, S4) and one sample (KB03) was from sediments initially presumed to be stratigraphically below the tracks (Figure S5). Samples KB01 and KB02 are currently >20m below the top of a 30m high cliff exposure next to the river (Figure S1). The samples have a clear stratigraphic relationship to the clay bed that the moa tracks were found in. Sample KB03 was collected from the modern river channel downstream of the tracks. The rocks were of unknown relationship to the Maniototo Conglomerate Formation mudstones found in the riverbed north of the bridge (Figures S5, S6), see further discussion below and in **main text**.


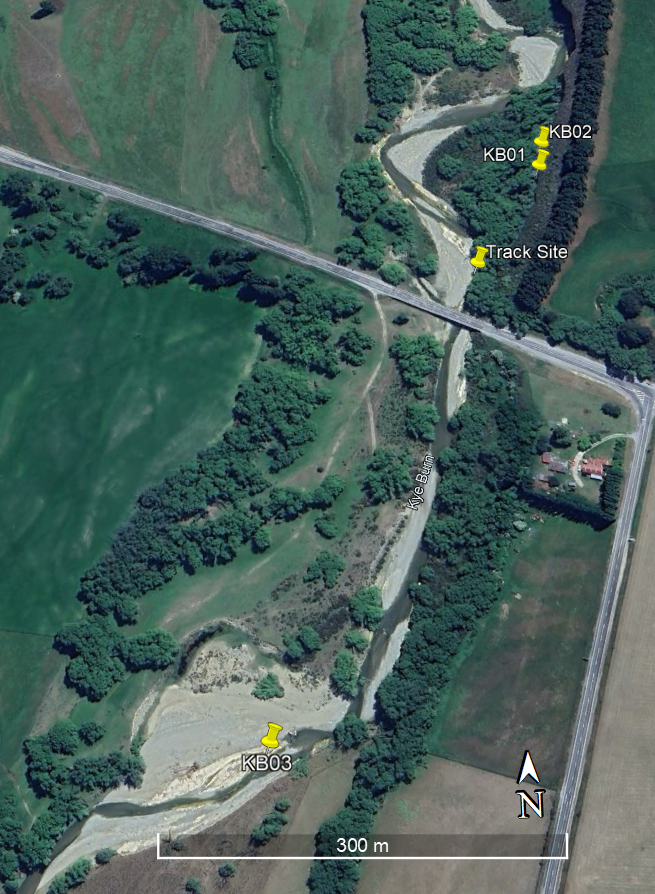


Figure S1: Locations of cosmogenic nuclide samples.


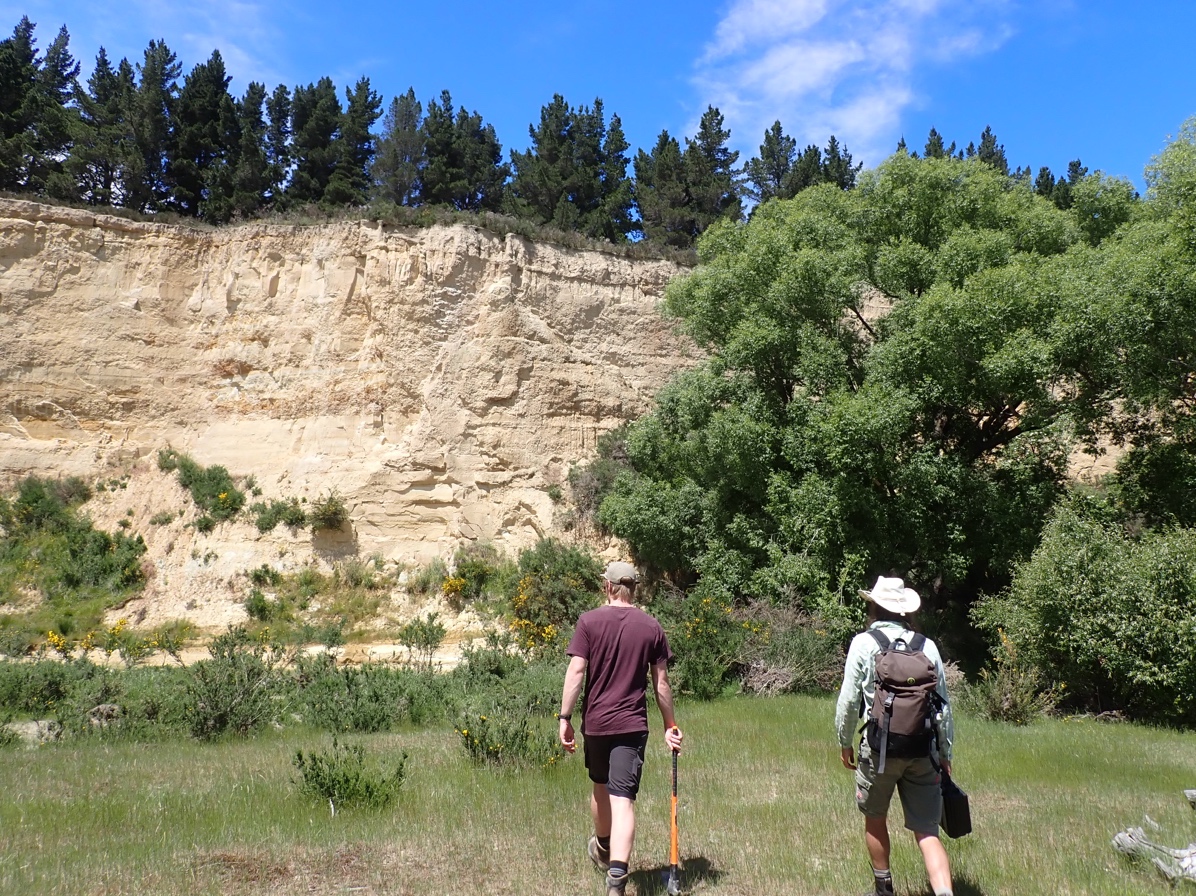


Figure S2: The 30 m high cliff of indurated Maniototo Conglomerate, Kyeburn River east bank, 200 m upstream from the moa trackway site. Source of samples KB01 and KB02. Note laterally continuous beds of fine grained sediments with faint bedding; conglomerates prevalent upsequence.


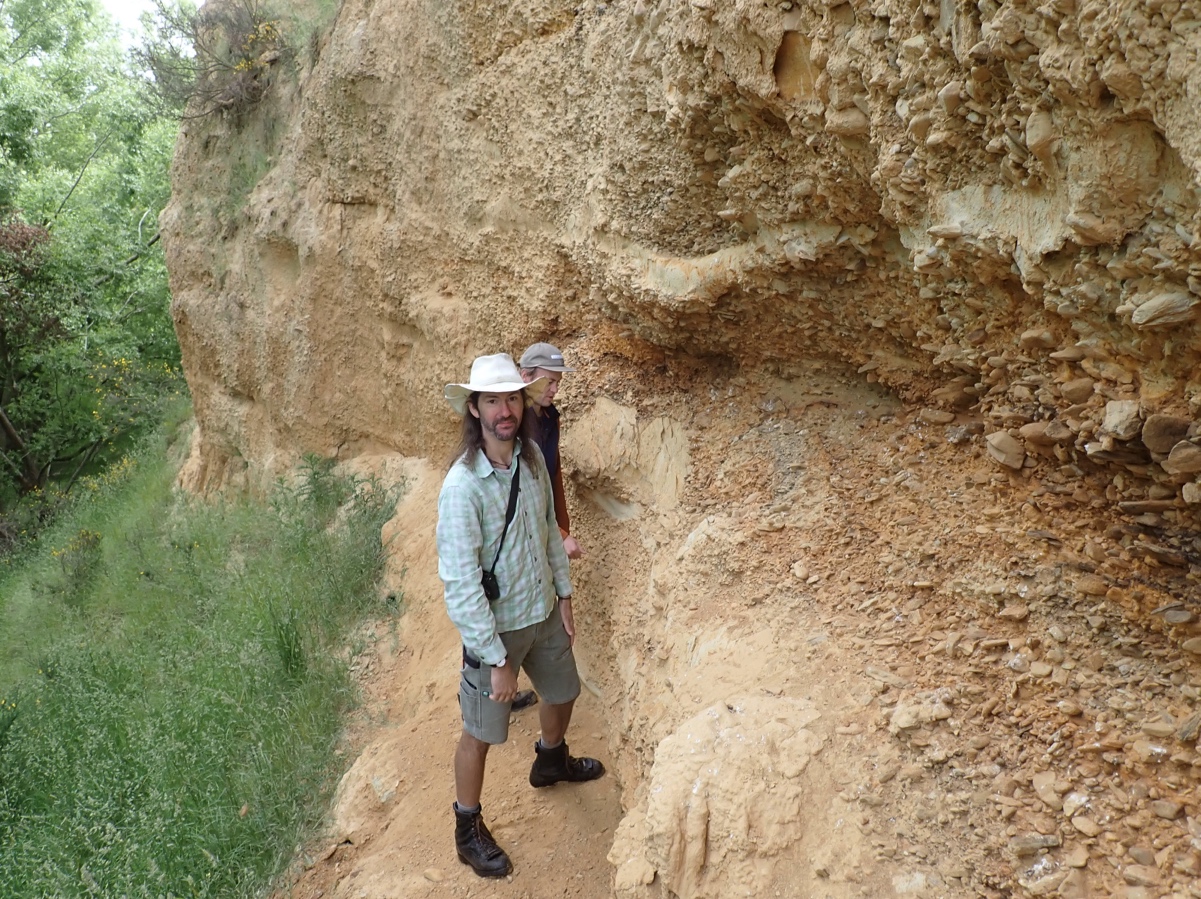


Figure S3: Source conglomerate for KB-01 in Maniototo Conglomerate Formation. Kevin Norton (left) and Kane Fleury (right). Field photos by Marcus Richards on 10^th^ December,2021.


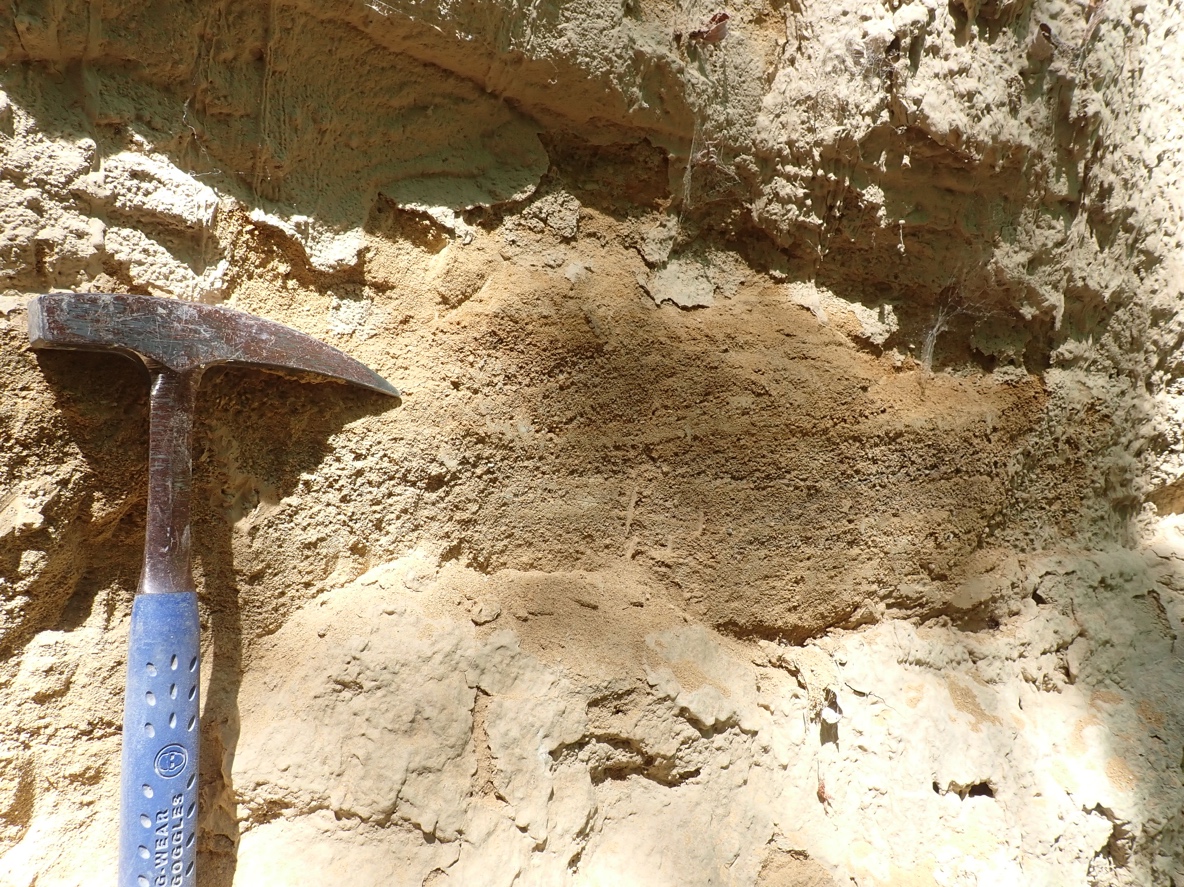


Figure S4: Source sandstone for KB-02 in Maniototo Conglomerate Formation.


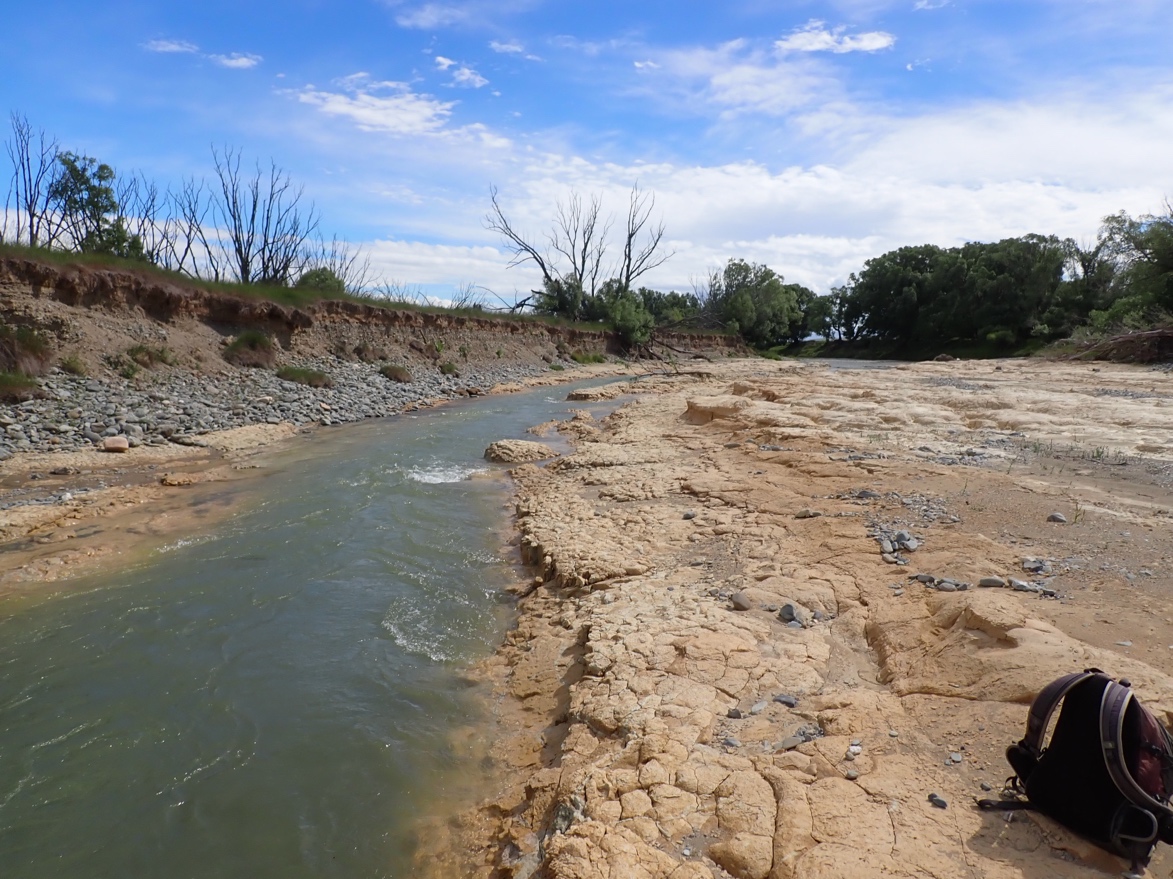


Figure S5: Source sandstone for KB-03, ~100 m downstream of moa trackway site. Note different texture to outcrops in Figure S6. Thought to be a later Pleistocene river deposit.


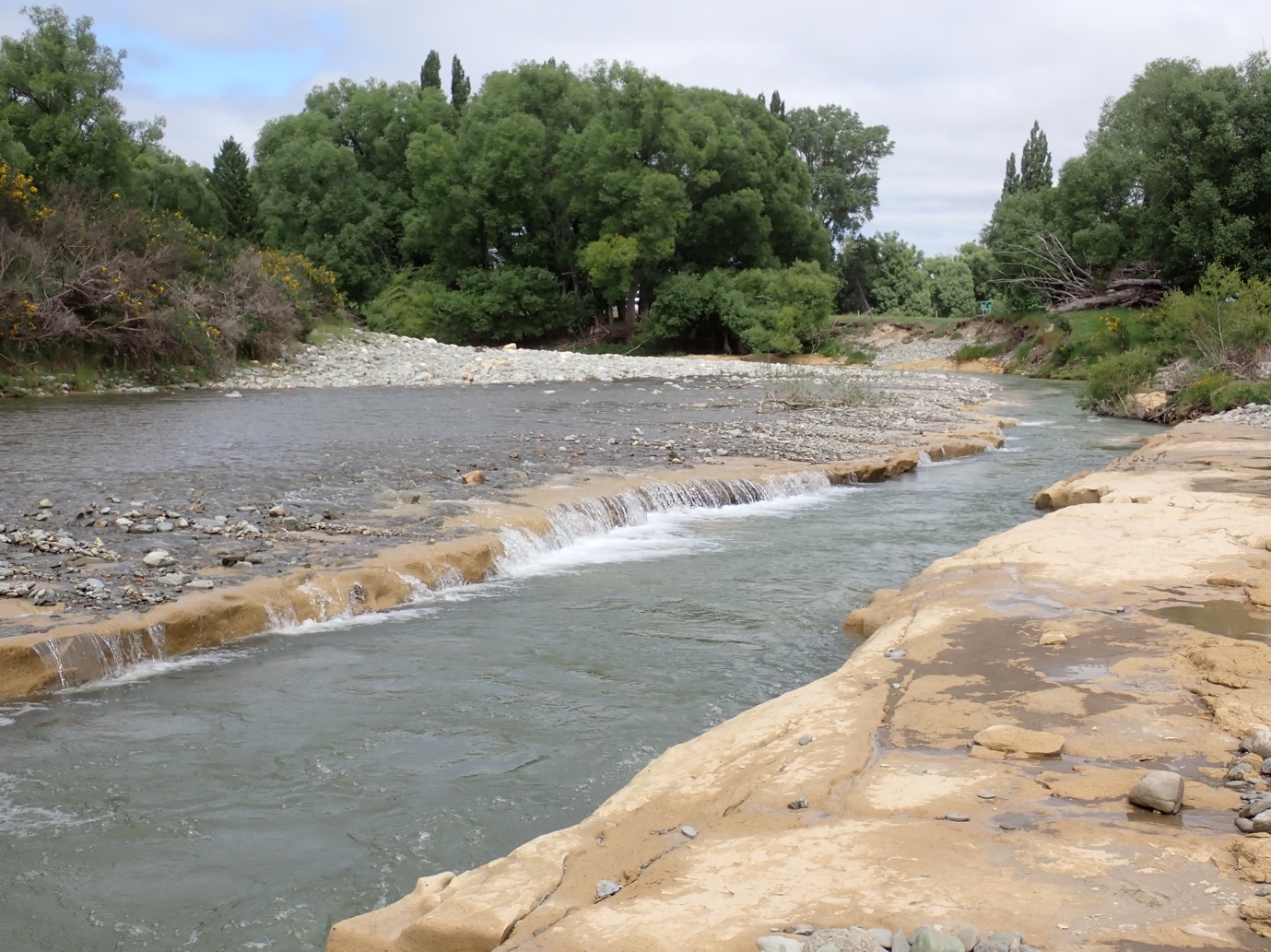


Figure S6: The massive indurated mudstones of Maniototo Conglomerate Formation being carved by the river. These outcrops are found in the Kyeburn river bed for the >200 m of riverbed upstream of and including the outcrop of the moa trackway site.

The samples were initially processed to clean quartz at the VUW Cosmogenic Nuclide Laboratory. Subsequent extraction of 26Al and 10Be as well as AMS isotope ratio measurements were conducted at ANSTO. Samples KB01 and KB02 yield similar 10Be and 26Al concentrations with KB03 having significantly lower concentrations (Table S1). When plotted on a 26Al/10Be vs 10Be plot, all samples indicate burial (Figure S7). KB01 and KB02 cluster while KB03 plots towards lower 10Be values, potentially indicating different original source areas.

Table S1: Inputs for cosmogenic nuclide calculator.


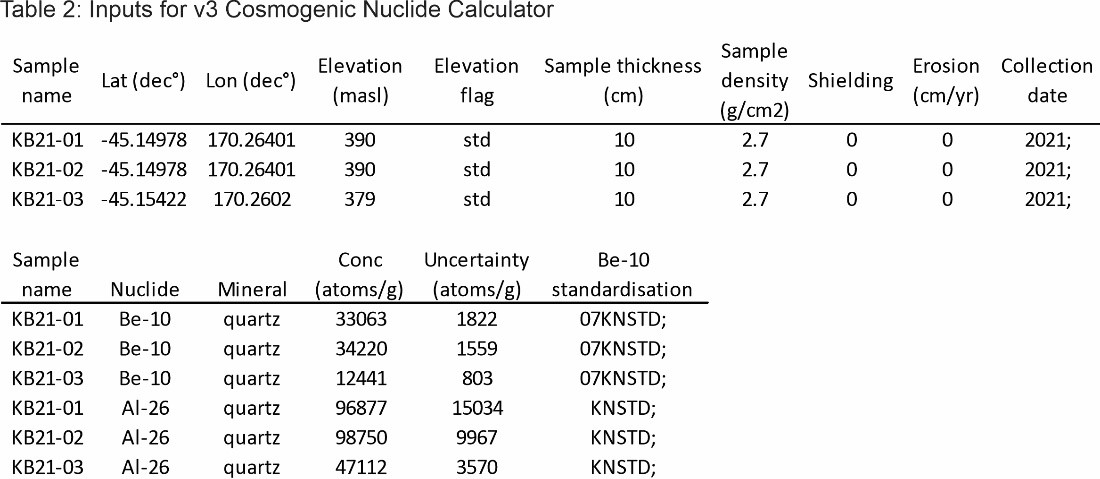


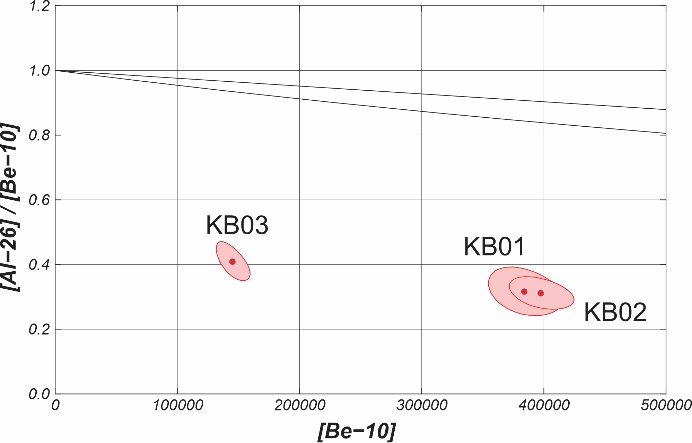


Figure S7: Multi-nuclide plot of samples. Upper sloping lines are the constant exposure lines. Samples plotting below those lines indicates burial.

The simple burial ages (i.e. assuming infinite burial depth and an initial 26Al/10Be ratio equal to the modern production ratio. The modern production ratio of 7.01 was derived from the production rates for 26Al and 10Be calculated by v3 of the Balco online nuclide calculator) yielded burial ages of KB01 = 1.90(+0.48/-0.44)Ma, KB02 = 1.93(+0.33/-0.31)Ma, and KB03 = 1.34(+0.31/-0.30)Ma. Any burial time spent at shallower depths (i.e. where muon interactions produce cosmogenic nuclides) will result in an older burial age. Therefore, we can confidently assign a minimum age of ~1.9Ma to the Maniototo Conglomerate Formation north of the Kyeburn Bridge on State Highway 85.

Because we know the samples were collected from shallow depths (~20m for KB01 and KB02, and ~50cm for KB03), we constructed a Monte Carlo model to model 26Al and 10Be concentrations through time assuming initial burial at any depth between the modern sample depth and 150m. The initial age of burial was allowed to vary between 10Ma and 1Ma. The initial 26Al and 10Be concentrations were calculated assuming steady state erosion of the sediment source area at an erosion rate between 0.001 and 0.01cm/yr following:

$$N=\frac{P_{0}}{(\lambda+\rho\times\frac{\varepsilon}{\Lambda})}$$

Where N is the steady state nuclide concentration (atoms/g), P_0_ is the nuclide production rate at the surface (atoms/g/yr; from v3 of the Balco online nuclide calculator), λ is the mean-life of the nuclide (yr), ρ is the density of the rock (g/cm^3^), ε is the input erosion rate (cm/yr), and Λ is the attenuation path length (g/cm^2^).

The nuclide concentration is then calculated through time using an iterative model (Figure S8). Production rate at depth includes production from both neutrons and stopped and fast muons following Schaller et al., 2002:

$$N={N\times{exp}^{-\lambda t}+P}_{nuc}0\times\sum_{i=1}^{2} \frac{a_{i}\times{exp}^{\left( \rho\times\frac{z}{b_{i}} \right)}\times\left( 1-{exp}^{-t\lambda} \right)}{\lambda}+P_{\mu stopped}0\times\sum_{j=1}^{3} \frac{a_{j}\times{exp}^{(\rho\times\frac{z}{b_{j}})}\times\left( 1-{exp}^{-t\lambda} \right)}{\lambda}+P_{\mu fast}0\times\sum_{k=1}^{3} \frac{a_{k}\times{exp}^{(\rho\times\frac{z}{b_{k}})}\times\left( 1-{exp}^{-t\lambda} \right)}{\lambda}$$

Where a and b are scalars for production and attenuation derived from fitting the equations of Heisinger et al., 2002a and Heisinger et al., 2002b. Note that the initial nuclide ratio is dependent on the input erosion rate so that burial ages slightly younger than the simple burial ages above are possible for very slow erosion. The mean best fit ages for KB01 and KB02 (3.76Ma and 3.74Ma, respectively) are equivalent within error. There are no good fits for KB03 using the same erosion rates as for KB01 and KB02. However, if the erosion rate were ~3x faster during the erosion of the sediments that were eventually deposited as KB03, then the range of allowable burial ages (2.26 to 4.88Ma) are within error of KB01 and KB02. It is therefore possible that the sand layer from which KB03 was collected belongs to a different unit which came from a more rapidly eroding landscape.


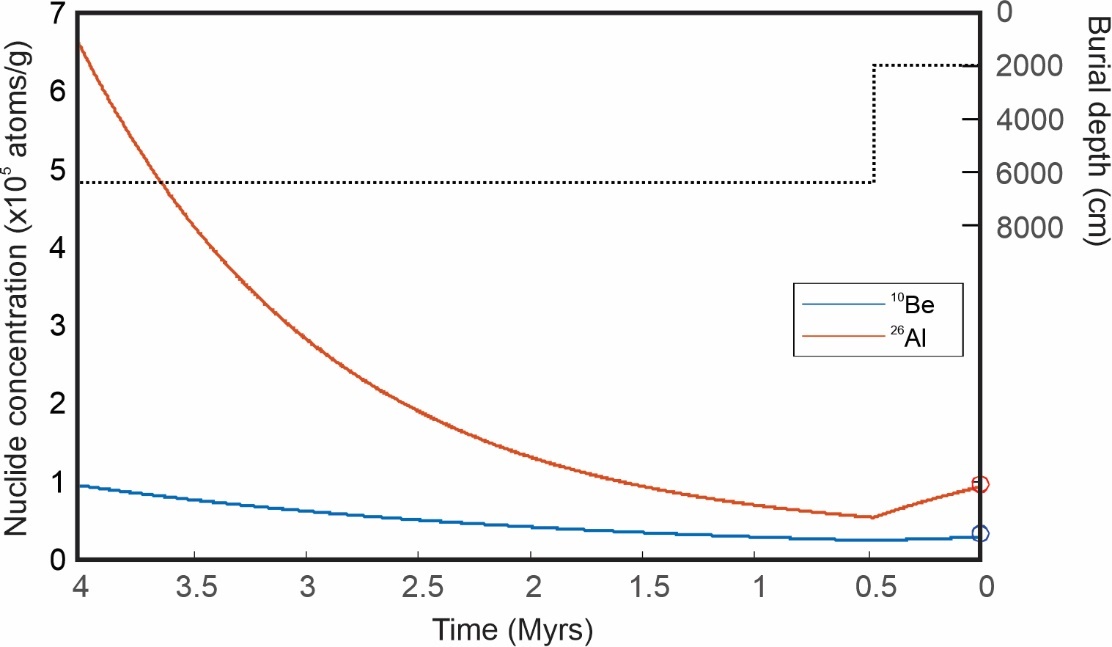


Figure S8: model run. The 26Al and 10Be concentrations are modelled through time as a function of burial depth. Models that fit the measured data within 1-sigma of the measurement error are deemed good fits.


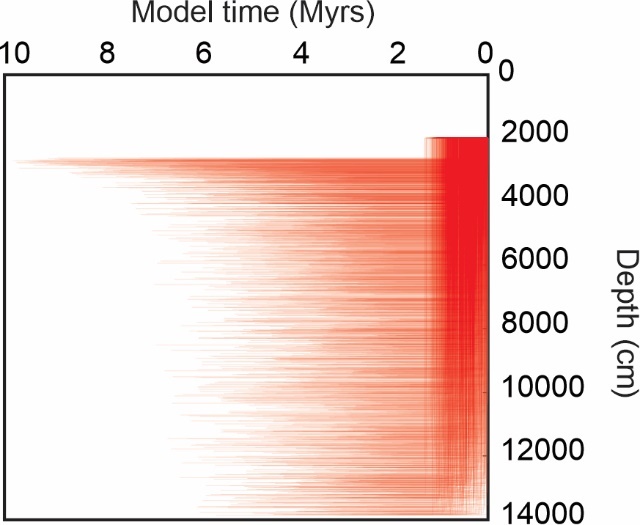


Figure S9: Best fit depth histories for KB01.

References:

Heisinger, B., Lal, D., Jull, A.J.T., Kubik, P.W., Ivy-Ochs, S., Neumaier, S., Knie, K., Lazarev, V., Nolte, E., 2002a. Production of selected cosmogenic radionuclides by muons: 1. Fast muons. Earth and Planetary Science Letters 200, 345–355.

Heisinger, B., Lal, D., Jull, A.J.T., Kubik, P.W., Ivy-Ochs, S., Knie, K., Nolte, E., 2002b. Production of selected cosmogenic radionuclides by muons: 2. Capture of negative muons. Earth and Planetary Science Letters 200, 357– 369.

Supplemental file 2: Model parameters for model.

P10_0 = 5.42; %atoms/g/yr 10Be

P26_0 = 38.04; %atoms/g/yr 26Al

tau10 = 1390000/log(2); %yr decay constant of 10Be

tau26 = 720000/log(2); %yr decay constant of 26Al

lambda10 = 1/tau10; %yr-1 mean life of 10Be

lambda26 = 1/tau26; %yr-1 mean life of 26Al

atten = 160; %g/cm2

rho = 2.6; %g/cm3

P10_0_n = P10_0 * 0.964008; %percent neutron contribution determined from Schaller et al., 2002

P10_0_sm = P10_0 * 0.019172; %percent stopped muon contribution determined from Schaller et al., 2002

P10_0_fm = P10_0 * 0.01682; %percent fast muon contribution determined from Schaller et al., 2002

P26_0_n = P26_0 * 0.964008;

P26_0_sm = P26_0 * 0.019172;

P26_0_fm = P26_0 * 0.01682;

ia1 = 1.0747; %exponential scalars from Schaller et al., 2002.

ib1 = 157;

ia2 = -0.0747;

ib2 = 5.887;

ja1 = -0.050;

jb1 = 160;

ja2 = 0.845;

jb2 = 1030;

ja3 = 0.205;

jb3 = 3000;

ka1 = 0.010;

kb1 = 100;

ka2 = 0.615;

kb2 = 1520;

ka3 = 0.375;

kb3 = 7600;
