# Supplementary material for: The moa footprints from the Pliocene – early Pleistocene of Kyeburn, Otago, New Zealand
Source: J R Soc N Z. 2023 Nov 14;54(5):620–42. doi: 10.1080/03036758.2023.2264789 (PMC11459810; doi:10.1080/03036758.2023.2264789)
Supplement: Supplemental File 2 data [file TNZR_A_2264789_SM6177.pdf]

Museum_code	Specimen_number	Notes	Family	Genus	Species	Genus_species	PL_or_PR	Digit2_length	Total_length_of_toe_to_heel-digit2	Width_of_digit2	Digit3_length	Total_length_of_toe_to_heel_digit3 (Total footprint length)	Width_of_digit3	Digit4_length	Total_length_of_toe_to_heel-digit4	Width_of_digit4	distal_TMT_width	Depth of impression (if footprint)
OMNZ	AV11896a	Print badly eroded at caudal margins	TBC	TBC	TBC	Kyeburn footprint L1	PL	95	210	39	170	250	40	115	210	38		37
OMNZ	AV11896b		TBC	TBC	TBC	Kyeburn footprint R1	PR	110	199	50	161	260	43	112	205	53	116	51
OMNZ	AV11896c		TBC	TBC	TBC	Kyeburn footprint L2	PL	99	177	48	175	278	40	120	217	34	101	44
OMNZ	AV11896d		TBC	TBC	TBC	Kyeburn footprint R2	PR	105	192	41	190	294	45	124	215	40	103	44
OMNZ	AV11896e		TBC	TBC	TBC	Kyeburn footprint L3	PL	109	186	49	179	281	46	137	223	42	99	50
OMNZ	AV11896f	Digit2 distal end extends 21mm into the block, the measurement of digit length includes this.	TBC	TBC	TBC	Kyeburn footprint R3	PR	105	185	45	170	280	44	119	210	44	108	52
OMNZ		Captured in imagry only	TBC2	Dinornis	TBC2	Kyeburn2	TBC (left suspected)	170	266	66	205	285	58	168	283	55	101	
OMNZ	AV5991		Emeidae	Euryapteryx	curtus	Euryapteryx curtus	PL	126		32.5	152		32	111.3		27	108	
OMNZ	AV5993		Dinornithidae	Dinornis	robustus	Dinornis robustus (male)	PL	177.5		36	233		40.5	172		37	141	
OMNZ	AV3816		Dinornithidae	Dinornis	robustus	Dinornis robustus (female)	PR	175		42	222		44	217		40	157	
OMNZ	AV3845		Emeidae	Euryapteryx	curtus	Euryapteryx curtus	PL	117		30	155		34	120		28	107	
OMNZ	AV5992		Emeidae	Pachyornis	elephantopus	Pachyornis elephantopus	PR	124		35	173		39	113		34	123	
OMNZ	AV6039		Emeidae	Anomalopteryx	didiformis	Anomalopteryx didiformis	PR	118		21.5	164		24	146		21	70	
OMNZ	AV5379		Dinornithidae	Dinornis	robustus	Dinornis robustus (male)	PL	152		32	169		32.5	154		30	116	
OMNZ	AV5379	SLSP	Dinornithidae	Dinornis	robustus	Dinornis robustus (male)	PR	140		31	185		35.5	164		29	116	
OMNZ	AV5374	SLSP	Dinornithidae	Dinornis	robustus	Dinornis robustus (female)	PL	203		42	251		44	209		41	152	
OMNZ	AV5374	SLSP	Dinornithidae	Dinornis	robustus	Dinornis robustus (female)	PR	205		41	257		44	206		41	152	
OMNZ	AV5370		Emeidae	Pachyornis	elephantopus	Pachyornis elephantopus	PL	151		41	214		49	155		40	136	
OMNZ	AV5370		Emeidae	Pachyornis	elephantopus	Pachyornis elephantopus	PR	156		40	207		49	157		35	136	
OMNZ	AV8324	Left foot missing the 3rd distal phalage bone. Digit 2, middle and anterieor digit is fused so measurement slightly less accurate.	Emeidae	Euryapteryx	curtus	Euryapteryx curtus	PL	115		27	143		32			26	105	
OMNZ	AV4805	Partially complete, middle toe only	Emeidae	Pachyornis	elephantopus	Pachyornis elephantopus	PR			34	151		40			34	120	
OMNZ	AV5372		Emeidae	Pachyornis	elephantopus	Pachyornis elephantopus	PL	133		31.5	175		34	123		31.5	128	
OMNZ	AV5372		Emeidae	Pachyornis	elephantopus	Pachyornis elephantopus	PR	130		29	179		35.5	121		32	120	
OMNZ	AV5371		Emeidae	Emeus	crassus	Emeus crassus	PL	98		25.5	145		29	105		26	90	
OMNZ	AV5371	Outside digits are pretty good. Middle digit missing the claw only.	Emeidae	Emeus	crassus	Emeus crassus	PR	104		26			26.5	111		26	98	
OMNZ	AV5377		Emeidae	Pachyornis	elephantopus	Pachyornis elephantopus	PL	142		35	174		35	120		32.5	121	
OMNZ	AV5377		Emeidae	Pachyornis	elephantopus	Pachyornis elephantopus	PR	144		34	177		35	129		29	119	
OMNZ	AV9031		Dinornithidae	Dinornis	robustus	Dinornis robustus (female)	PL	184		41	223		44	195		38.5	157	
OMNZ	AV9031		Dinornithidae	Dinornis	robustus	Dinornis robustus (female)	PR	180		42	227		45	209		38.5	161	
OMNZ	AV10049	Left foot missing most of the bones and the TMT so not included in dataset. The right foot has larger than ideal gaps in the bones which will overestimate slightly the length of the foot of this individual. It is also missing bones from the 4th digit.	Megalapterygidae	Megalapteryx	didinus	Megalapteryx didinus	PR	137		22.5	176		26			23.5	88	
Southland	85.282	Digit IV missing, articulated	Emeidae	Anomalopteryx	didiformis	Anomalopteryx didiformis	PR	93		23	127		24.5				89.5	
Southland	E80.4	Mummified lower leg, missing distal phlanges from digit 3, skin covers the measurements as follows: digit 2 width skin both sides, digit 3 width skin on one side, digit 4 skin on both sides, TMT skin on outer side only, there is appoximately 4.5mm of skin on each side of the thickest point of the toe bones.	Emeidae	Anomalopteryx	didiformis	Anomalopteryx didiformis	PL	143		25			26.5	138		24	81	
Southland	A47.32	Some bones have come out of the articualtion digit 4 length than recorded due to articulation. Digit 4 length is approximate and estimated due to incompleteness	Dinornithidae	Dinornis	robustus	Dinornis robustus (female)	PL	211		40	262		42.5	230		40	164	
Southland	A41.15	Digit 4 phalanges articulated in reverse.	Emeidae	Emeus	crassus	Emeus crassus	PL	112		20	146		23	110		21	81	
Southland	A41.15		Emeidae	Emeus	crassus	Emeus crassus	PR	109		20	147		25	104		22.5	87.5	
Waitaki Museum		End of d3 and d4 claws are broken off and not present. About 5mm-10mm from the tips of both digits absent.	Emeidae	Emeus	crassus	Emeus crassus	PL	123		27	149		31	145		27.5	96	
